# Synthesis and Evaluation of a Fluorine-18 Radioligand for Imaging Huntingtin Aggregates by Positron Emission Tomographic Imaging

**DOI:** 10.3389/fnins.2021.766176

**Published:** 2021-12-02

**Authors:** Tanpreet Kaur, Allen F. Brooks, Alex Lapsys, Timothy J. Desmond, Jenelle Stauff, Janna Arteaga, Wade P. Winton, Peter J. H. Scott

**Affiliations:** Division of Nuclear Medicine, Department of Radiology, University of Michigan Medical School, Ann Arbor, MI, United States

**Keywords:** Huntington’s disease, PET imaging, radiotracer, fluorine-18, [^11^C]CHDI

## Abstract

Mutations in the huntingtin gene (HTT) triggers aggregation of huntingtin protein (*m*HTT), which is the hallmark pathology of neurodegenerative Huntington’s disease (HD). Development of a high affinity ^18^F radiotracer would enable the study of Huntington’s disease pathology using a non-invasive imaging modality, positron emission tomography (PET) imaging. Herein, we report the first synthesis of fluorine-18 imaging agent, 6-(5-((5-(2,2-difluoro-2-(fluoro-^18^F)ethoxy)pyridin-2-yl)methoxy)benzo[*d*]oxazol-2-yl)-2-methylpyridazin-3(2*H*)-one ([^18^F]1), a radioligand for HD and its preclinical evaluation *in vitro* (autoradiography of post-mortem HD brains) and *in vivo* (rodent and non-human primate brain PET). [^18^F]1 was synthesized in a 4.1% RCY (decay corrected) and in an average molar activity of 16.5 ± 12.5 GBq/μmol (445 ± 339 Ci/mmol). [^18^F]1 penetrated the blood-brain barrier of both rodents and primates, and specific saturable binding in post-mortem brain slices was observed that correlated to *m*HTT aggregates identified by immunohistochemistry.

## Introduction

**FIGURE 1:**
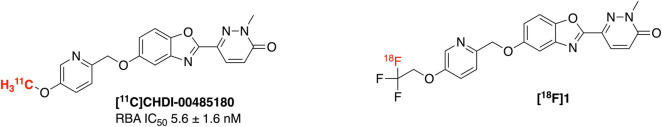
[^11^C]CHDI and [^18^F]1 for PET imaging of *m*HTT.

Huntington’s disease (HD) (Jenkins and Conneally, 1989; Bhattacharyya, 2016) is a neurodegenerative disease that progressively damages the motor, cognitive and psychiatric functions of patients (Dominguez and Munoz-Sanjuan, 2014; Cybulska et al., 2020). There is currently no approved therapy capable of delaying or slowing down HD onset or its progression (Estevez-Fraga et al., 2020). HD is primarily caused by the mutation in a single autosomal dominant gene leading to the formation of the mutant huntingtin gene (*m*HTT). *m*HTT with expanded CAG trinucleotide repeats (>36 CAG), encodes elongated polyQ repeats in the *N*-terminus, which in turn triggers aggregation of the huntingtin protein (Moldovean and Chiş, 2020). The pathology of HD is characterized by huntingtin protein aggregates. Although normal HTT is expressed throughout the body, the *m*HTT selectively targets brain cells and results in deteriorating medium spiny neurons of the striatum and cortex regions (DiFiglia, 2020). Design of suitable imaging agents for quantification of *m*HTT using positron emission tomography (PET) imaging will fill a critical gap in HD research by enabling non-invasive identification and tracking of huntingtin protein aggregates. PET imaging is a highly sensitive and noninvasive technique for quantifying biological targets within a living human and enabling their use as biomarkers of a disease. Thus a PET imaging agent for *m*HTT could be expected to have similar benefits to the amyloid, tau and α-synuclein PET imaging agents currently used for dementia imaging (Mathis et al., 2017), allowing diagnosis of HD, monitoring of disease progression, and evaluating patient response to HD modifying therapies targeting *m*HTT. The only example to date for imaging *m*HTT is with the ^11^C-labeled agents [^11^C]CHDI-180R and [^11^C]CHDI-626 which exhibit high affinity (low nanomolar IC_50_) toward *m*HTT and have selectivity over other protein aggregates like amyloid and tau (Dominguez et al., 2016; Liu et al., 2020, 2021; Bertoglio et al., 2021). To the best of our knowledge, there are currently no ^18^F-labeled PET imaging agents for imaging huntingtin protein aggregates described in the literature. Driven by the remarkable results achieved by [^11^C]CHDI series in HD imaging, herein we describe the first synthesis of an ^18^F analog (**[^18^F]1**) to image *m*HTT in HD patients (Figure 1).

**FIGURE 2:**
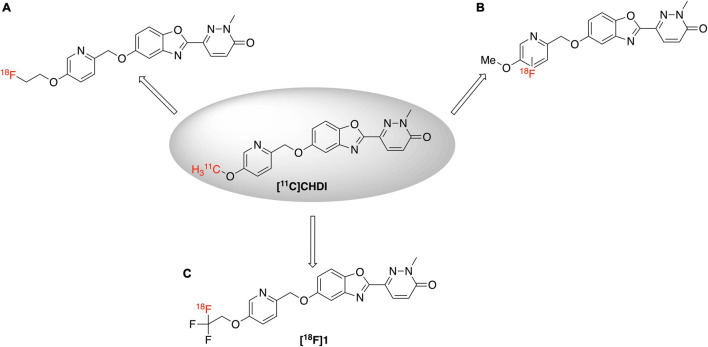
Proposed ^18^F analogs of [^11^C]CHDI for PET imaging of *m*HTT.

An ^18^F-labeled analog is highly desirable because of the longer half-life of fluorine-18 (109.8 min) (Ross and Wester, 2011) compared to carbon-11 (20.4 min), which will enable its use in longer imaging studies to improve signal to background in the image, facilitate more sophisticated imaging studies with blocking agents, and eventually distribution to off-site PET imaging facilities. Looking at the structure of [^11^C]CHDI-180R, we chose not to incorporate ^18^F either as the 2-fluoroethyl group (Figure 2A) due to potential metabolic instability and generation of toxic side products (Pan, 2019), nor on the pyridine ring due to undesirable stereoelectric effects that could affect the imaging agents binding to huntingtin aggregates (Figure 2B). So, we decided to incorporate ^18^F on the CHDI scaffold as a trifluoromethyl group (Figure 2C) to limit the potential for metabolic instability and to avoid negatively impacting target engagement.

The trifluoromethyl (CF_3_) group is a common motif in small molecule-based drug scaffolds (Yale, 1959; Lien and Riss, 2014). Moreover, 2-[^18^F]trifluoromethyl groups not only acts as a prosthetic group but also have improved metabolic stability when compared to 2-fluoromethyl groups, while still providing a similar straightforward means to incorporate fluorine-18 into drug scaffolds for PET imaging (Riss et al., 2012). Installation of trifluoromethyl groups on drug scaffolds serves as a common way of lead optimization in drug development to improve metabolic stability and overall pharmacokinetics. The strong electron withdrawing nature and higher stability of CF_3_ groups mean they have also received widespread interest from PET radiochemists, resulting in the development of a variety of novel labeling methods (Chen et al., 2015; Taddei et al., 2021). The synthesis of CF_3_ groups can be achieved by electrophilic fluorination (Chirakal et al., 1995; Teare et al., 2007), isotopic exchange reactions (Suehiro et al., 2011), nucleophilic fluorination (Kramer et al., 2020), transition metal catalyzed reactions (Huiban et al., 2013) and other methods (Chen et al., 2015). By far, the most common and preferred route is to carry out nucleophilic fluorination by [^18^F]fluoride ion (Ross and Wester, 2011). The required difluorovinyl-functionalized labeling precursors (Rafique et al., 2018) can be predominantly assessed by numerous synthetic routes e.g., CH activation and elimination (Yang et al., 2020), Wittig reaction (Fuqua et al., 1965; Li et al., 2017), Julia-Kocienski-type reactions (Burton et al., 1996; Zhao et al., 2010; Zheng et al., 2013) and other methods (Ichikawa et al., 1991). Herein, we describe synthesis of desired [^18^F]trifluoroethyl product **[^18^F]1** through a nucleophilic addition of H[^18^F]F to a difluorovinyl-functionalized precursor. We also report the preclinical evaluation of **[^18^F]1** *in vitro* (binding affinity experiments, and saturation binding autoradiography experiments with post-mortem HD brain tissue samples), and *in vivo* (rodent and nonhuman primate (NHP) PET imaging).

## Materials and Methods

### Chemistry

#### General Considerations

Unless otherwise stated all the chemicals were purchased from commercial suppliers and used without purification. Automated flash chromatography was performed with a Biotage Isolera Prime system. High-performance liquid chromatography (HPLC) was performed using a Shimadzu LC-2010A HT. ^1^H and ^13^C NMR spectra were collected on a Varian 500 NMR (500 MHz for ^1^H NMR and 125 MHz for ^13^C NMR), in DMSO-d_6_ or CDCl_3_ unless otherwise indicated, δ in ppm rel. to tetramethylsilane (δ = 0), J in Hz. Mass spectra were measured on an Agilent Q-TOF HPLC-MS.

#### Synthesis of Standard 1 and Precursor 11

**5-(2,2,2-Trifluoroethoxy)pyridin-2-yl)methanol (3):** 6-(Hydroxymethyl)pyridin-3-ol (**2**, 400 mg, 3.2 mmol) was dissolved in anhydrous DMF (5 mL) and K_2_CO_3_ (1.3 g, 9.6 mmol) was added to the solution. The reaction mixture was stirred at room temperature for 15 min. Trifluoroethyl triflate (460 mg, 3.2 mmol) was added into the reaction mixture, and the resulting mixture was heated at 120°C for 24 h. Upon completion of the reaction, the mixture was cooled to room temperature, filtered to remove solids and quenched with sat. NH_4_Cl (5 mL). The quenched filtrate was extracted with ethyl acetate (EA) (3 × 50 mL), washed with H_2_O and dried over anhydrous Na_2_SO_4_. The crude reaction mixture was purified with silica gel flash chromatography using a hexane:EA gradient mobile phase. Product collected in 41% yield as a brown oil. ^1^H NMR (500 MHz; CDCl_3_)/δ (ppm): 8.25 (t, *J* = 1.8 Hz, 1H), 7.27 (d, *J* = 1.8 Hz, 2H), 4.69 (s, 2H), 4.38 (q, *J* = 8.0 Hz, 2H).^13^C NMR (125 MHz; CDCl_3_)/δ (ppm): 153.81, 152.91, 136.52, 126.30, 124.09, 123.34, 121.87, 121.42, 66.69, 66.40, 66.11, 65.83, 63.98.^19^F NMR (470 MHz, CDCl_3_)/δ (ppm):–73.99 (t, *J* = 8.3 Hz). HRMS: Calculated for [M+H]^+^ (M = C_8_H_9_F_3_NO_2_) = 208.0575, actual *m/z* = 208.0585.

**(5-((2,2-Difluorovinyl)oxy)pyridin-2-yl)methanol (4):** (5-(2,2,2-Trifluoroethoxy)pyridin-2-yl)methanol (235.5 mg, 1.13 mmol) was added to a flame-dried round bottom flask. Anhydrous THF (5 mL) was added into the flask and the resulting solution was cooled to –78°C over 15 min in a dry ice acetone bath. *n*-Butyl lithium in hexane (*n*-BuLi 1.6 M, 2.83 mL, 4.52 mmol) was added slowly to the reaction mixture and was stirred at the same temperature for 45 min. The reaction was quenched with THF:water (1:1, 5 mL) and warmed to room temperature. It was extracted with ethyl acetate (3 x 50 mL) and the combined organic fractions were rinsed with brine and dried over anhydrous Na_2_SO_4_. The crude reaction mixture was purified with silica gel flash chromatography using an EA:Hexane mobile phase gradient. The purified product was collected in 19% yield as a brown oil. ^1^H NMR (500 MHz; CDCl_3_)/δ (ppm): δ 8.30 (d, *J* = 2.9 Hz, 1H), 7.33 (dd, *J* = 8.6, 2.9 Hz, 1H), 7.30–7.25 (m, 1H), 6.10 (dd, *J* = 14.8, 3.5 Hz, 1H), 4.71 (s, 2H). ^13^C NMR (125 MHz; CDCl_3_)/δ (ppm): δ 159.13, 156.90, 156.81, 154.58, 154.14, 152.69, 136.68, 121.34, 104.61, 104.48, 104.16, 104.04. ^19^F NMR (470 MHz, CDCl_3_)/δ (ppm): δ–95.39,–95.42,–95.52,–95.55,–113.85 (d, *J* = 3.4 Hz),–113.98 (d, *J* = 3.6 Hz). HRMS: Calculated for [M+H]^+^ (M = C_8_H_8_F_2_NO_2_) = 188.0511, actual *m/z* = 188.0523.

**2-Methyl-5-((5-(2,2,2-trifluoroethoxy)pyridin-2-yl)methoxy)benzo[*d*]oxazole (5):** Alcohol **3** (118.7 mg, 0.57 mmol) and 2-methyl-1,3-benzoxazol-5-ol (77.3 mg, 0.52 mmol) were dissolved in anhydrous toluene (2 mL). Cyanomethylenetributylphosphorane (205 μL, 0.78 mmol) was then added and the resultant reaction mixture was heated at 100°C for 4 h. Upon completion of the reaction, toluene was evaporated using a rotary evaporator at reduced pressure. The crude mixture was triturated with diethyl ether/hexane (1:1, 4 mL) and the resultant solid product was filtered to isolate. Product was collected in 73% yield as a brown solid. ^1^H NMR (500 MHz; CDCl_3_)/δ (ppm): 8.34 (d, *J* = 2.5 Hz, 1H), 7.51 (d, *J* = 8.7 Hz, 1H), 7.36–7.24 (m, 2H), 7.20–7.10 (m, 1H), 6.90 (ddd, *J* = 57.8, 8.8, 2.5 Hz, 1H), 5.18 (s, 2H), 4.40 (q, *J* = 7.9 Hz, 2H), 2.59 (s, 3H).^13^C NMR (125 MHz; CDCl_3_)/δ (ppm): 64.95, 164.76, 155.63, 153.99, 153.11, 150.93, 145.88, 145.21, 142.07, 141.97, 136.98, 124.04, 123.21, 122.45, 121.82, 113.31, 113.14, 110.45, 110.29, 105.09, 104.21, 70.71, 66.61, 66.32, 66.04, 65.75, 14.54, 14.50. ^19^F NMR (470 MHz, CDCl_3_)/δ (ppm):-73.89 (t, *J* = 8.6 Hz). HRMS: Calculated for [M+H]^+^ (M = C_1__6_H_1__4_F_3_N_2_O_3_) = 339.0951, actual *m/z* = 339.0957.

**5-((5-((2,2-Difluorovinyl)oxy)pyridin-2-yl)methoxy)-2-methylbenzo[*d*]oxazole (6):** (5-((2,2-Difluorovinyl)oxy) pyridin-2-yl)methanol **4** (90 mg, 0.49 mmol) and 2-methyl-1,3-benzoxazol-5-ol (67.4 mg, 0.45 mmol) was dissolved in anhydrous toluene (2 mL), and cyanomethylenetributylphosphorane (177.9 μL, 0.68 mmol) was then added and the resultant reaction mixture was heated at 100°C for 4 h. Upon completion of the reaction, toluene was evaporated using a rotary evaporator at reduced pressure. The crude mixture was triturated with diethyl ether/hexane (1:1, 4 mL) and the resultant solid product was filtered to isolate. Product was collected in 54% yield as a brown solid.^1^H NMR (500 MHz; CDCl_3_)/δ (ppm): 8.44 (d, *J* = 2.9 Hz, 1H), 7.72 (d, *J* = 8.6 Hz, 1H), 7.60 (dd, *J* = 8.7, 2.8 Hz, 1H), 7.38 (d, *J* = 8.8 Hz, 1H), 7.23 (d, *J* = 2.6 Hz, 1H), 7.01 (dd, *J* = 8.8, 2.6 Hz, 1H), 6.17 (dd, *J* = 14.4, 3.7 Hz, 1H), 5.38 (s, 2H), 2.62 (s, 3H). ^13^C NMR (125 MHz; CDCl_3_)/δ (ppm): ^19^F NMR (470 MHz, CDCl_3_)/δ (ppm)-93.31, 111.70; HRMS: Calculated for [M+H]^+^ (M = C_1__6_H_1__2_F_2_N_2_O_3_) = 319.0894, actual *m/z* = 319.0882.

**2-Amino-4-((5-(2,2,2-trifluoroethoxy)pyridin-2-yl) methoxy)phenol (7):** 2-Methyl-5-((5-(2,2,2-trifluoroethoxy) pyridin-2-yl)methoxy)benzo[*d*]oxazole **5** (100 mg, 0.29 mmol) was dissolved in ethanol (10 mL) and in conc. HCl (2.0 mL). The reaction mixture was heated at 70 °C for 16 h. The solvent was evaporated, and crude reaction mixture was used directly for the next reaction. Product collected in 100% yield. ^1^H NMR (500 MHz; CDCl_3_)/δ (ppm): 8.74 (d, *J* = 2.8 Hz, 1H), 8.38–8.32 (m, 1H), 8.15 (d, *J* = 9.0 Hz, 1H), 7.17 (d, *J* = 3.0 Hz, 1H), 7.11 (dd, *J* = 9.0, 3.0 Hz, 1H), 7.01 (s, 1H), 5.42 (s, 2H), 4.93 (d, *J* = 8.2 Hz, 2H). ^13^C NMR (125 MHz; CDCl_3_)/δ (ppm): 132.29, 130.26, 126.72, 116.53, 111.30, 66.25, 48.09, 47.92, 47.89, 47.75, 47.58, 47.41, 47.24, 47.07. ^19^F NMR (470 MHz, CDCl_3_)/δ (ppm):–75.51 (t, *J* = 8.1 Hz). HRMS: Calculated for [M+H]^+^ (M = C_1__4_H_1__4_F_3_N_2_O_3_) = 315.0957, actual *m/z* = 315.0944.

**2-Amino-4-((5-((2,2-difluorovinyl)oxy)pyridin-2-yl) methoxy)phenol (8):** 5-((5-((2,2-Difluorovinyl)oxy)pyridin-2-yl)methoxy)-2-methylbenzo[*d*]oxazole **6** (82.5 mg, 0.25 mmol) was dissolved in ethanol (10 mL) and in conc. HCl (2.0 mL). The reaction mixture was heated at 70 °C for 16 h. The solvent was removed under reduced pressure, and the crude reaction mixture was used directly in the next reaction. Product was collected in 100% yield as a brown solid. HRMS: Calculated for [M+H]^+^ (M = C_1__4_H_1__2_F_2_N_2_O_3_) = 295.0894, actual *m/z* = 295.0881.

***N*-(2-Hydroxy-5-((5-(2,2,2-trifluoroethoxy)pyridin-2-yl)methoxy)phenyl)-1-methyl-6-oxo-1,6-di hydropyridazine-3-carboxamide (9):** 2-Amino-4-((5-(2,2,2-trifluoroethoxy) pyridin-2-yl)methoxy)phenol **7** (100 mg, 0.28 mmol) and 1-methyl-6-oxo-1,6-dihydropyridazine-3-carboxylic acid (53.9 mg, 0.35 mmol) were dissolved in pyridine (3.0 mL). 1-Ethyl-3-(3-dimethylaminopropyl)carbodiimide hydrochloride (66.9 mg, 0.35 mmol) was added to the reaction mixture and the reaction mixture was stirred at room temperature for 22 h. H_2_O (5 mL) was added and the precipitate was collected by filtration. The solid was thoroughly dried, redissolved in 7N ammonia in methanol (10 mL) and stirred for an hour. The solvent was removed to yield the title compound. Product was collected in 54% yield as an off-white solid. ^1^H NMR (500 MHz; DMSO)/δ (ppm): 10.01 (s, 1H), 9.56 (s, 1H), 8.40 (d, *J* = 2.9 Hz, 1H), 7.97 (d, *J* = 9.7 Hz, 1H), 7.94 (d, *J* = 3.0 Hz, 1H), 7.57 (dd, *J* = 8.7, 3.0 Hz, 1H), 7.51 (d, *J* = 8.7 Hz, 1H), 7.10 (d, *J* = 9.7 Hz, 1H), 6.84 (d, *J* = 8.8 Hz, 1H), 6.65 (dd, *J* = 8.8, 3.0 Hz, 1H), 5.04 (s, 2H), 4.89 (q, *J* = 8.8 Hz, 2H), 3.76 (s, 3H). ^13^C NMR (125 MHz; DMSO)/δ (ppm): 161.58, 159.21, 154.24, 151.84, 150.90, 142.44, 137.98, 137.20, 130.79, 129.75, 125.55, 122.03, 117.27, 110.41, 108.51, 74.87, 65.45, 65.18, 64.49, 64.05, 40.91.^19^F NMR (470 MHz, DMSO)/δ (ppm):–72.59 (d, *J* = 9.5 Hz). HRMS: Calculated for [M+H]^+^ (M = C_1__6_H_1__4_F_3_N_2_O_3_) = 451.1229, actual *m/z* = 451.1223.

***N*-(5-((5-((2,2-Difluorovinyl)oxy)pyridin-2-yl)methoxy)- 2-hydroxyphenyl)-1-methyl-6-oxo-1,6-dihydropyridazine-3-carboxamide (10):** 2-Amino-4-((5-((2,2-difluorovinyl) oxy)pyridin-2-yl)methoxy)phenol **8** (73.5 mg, 0.22 mmol) and 1-methyl-6-oxo-1,6-dihydropyridazine-3-carboxylic acid (42.9 mg, 0.28 mmol) were dissolved in pyridine (2.0 mL). 1-Ethyl-3-(3-dimethylaminopropyl)carbodiimide hydrochloride (53.1 mg, 0.28 mmol) was added into the reaction mixture and the reaction mixture was stirred at room temperature for 22 h. H_2_O (5 mL) was added and the precipitate was collected by filtration. The solid was thoroughly dried, redissolved in 7N ammonia in methanol (10 mL) and stirred for an hour. The solvent was removed to yield the title compound in 26% yield as an off-white solid. ^1^H NMR (500 MHz, CDCl_3_) /δ (ppm): 9.31 (s, 1H), 8.46–8.34 (m, 1H), 8.02 (dd, *J* = 9.6, 1.2 Hz, 1H), 7.67 (d, *J* = 8.5 Hz, 1H), 7.58 (d, *J* = 9.5 Hz, 1H), 7.38 (s, 1H), 7.03 (d, *J* = 9.6 Hz, 1H), 6.95 (d, *J* = 8.8 Hz, 1H), 6.71 (dd, *J* = 8.9, 2.9 Hz, 1H), 6.17 (dd, *J* = 14.3, 3.6 Hz, 1H), 5.25 (d, *J* = 5.5 Hz, 2H), 3.92 (d, *J* = 1.1 Hz, 3H). ^19^F NMR (470 MHz, CDCl_3_)/δ (ppm):–95.11,–95.14,–95.24,–95.27,–113.53. HRMS: Calculated for [M+H]^+^ (M = C_2__0_H_1__6_F_2_N_4_O_5_) = 431.1167, actual *m/z* = 431.1158.

**2-Methyl-6-(5-((5-(2,2,2-trifluoroethoxy)pyridin-2-yl) methoxy)benzo[d]oxazol-2-yl)pyridazine 3(2*H*)-one (1):** *N*-(2- hydroxy-5-((5-(2,2,2-trifluoroethoxy)pyridin-2-yl)methoxy) phenyl)-1-methyl-6-oxo-1,6-dihydropyridazine-3-carboxamide **9** (30 mg, 0.066 mmol) was dissolved in anhydrous toluene (9 mL) and *p*TsOH.H_2_O (38 mg, 0.2 mmol) was added. The reaction mixture was refluxed using a Dean Stark trap for 24 h. It was extracted with EA (3 x 50 mL), washed with H_2_O and dried over anhydrous Na_2_SO_4_. The crude reaction mixture was purified via silica gel flash chromatography using a DCM:MeOH mobile phase gradient. Product was collected in 80% yield as an off-white solid. ^1^H NMR (499 MHz, CD_3_OD) δ 8.65 (d, *J* = 3.5 Hz, 1H), 8.47 (d, *J* = 9.6 Hz, 1H), 7.95–7.78 (m, 3H), 7.63 (d, *J* = 2.5 Hz, 1H), 7.46 (dd, *J* = 9.0, 2.5 Hz, 1H), 7.41 (d, *J* = 9.7 Hz, 1H), 5.52 (s, 2H), 4.84 (t, *J* = 8.1 Hz, 2H), 4.24 (s, 3H). ^13^C NMR (125 MHz; CD_3_OD)/δ (ppm): 160.62, 157.81, 155.78, 153.69, 149.65, 144.78, 141.88, 136.54, 134.00, 131.17, 129.27, 123.94, 123.25, 115.98, 111.45, 104.44, 66.01, 65.93, 65.74, 40.69, 29.47.^19^F NMR (470 MHz, CD_3_OD)/δ (ppm): δ-70.77 (t, *J* = 8.2 Hz). HRMS: Calculated for [M+H]^+^ (M = C_2__0_H_1__6_F_3_N_4_O_4_) = 433.1124, actual *m/z* = 433.1113.

**6-(5-((5-((2,2-Difluorovinyl)oxy)pyridin-2-yl)methoxy) benzo[*d*]oxazol-2-yl)-2-methylpyridazin-3 (2*H*)-one (11):** N-(5-((5-((2,2-difluorovinyl)oxy)pyridin-2-yl)methoxy)-2- hydroxyphenyl)-1-methyl-6-oxo-1,6-dihydropyridazine-3-carboxamide **10** (12.9 mg, 0.03 mmol) was dissolved in anhydrous toluene (3 mL) and *p*-TsOH.H_2_O (17.1 mg, 0.09 mmol) was added. The reaction mixture was refluxed using a Dean Stark trap for 24 h. It was extracted with EA (3 x 50 mL), washed with H_2_O and dried over anhydrous Na_2_SO_4_. The crude reaction mixture was purified via silica gel flash chromatography using a DCM:MeOH mobile phase gradient. Product was collected in 74% yield as an off-white solid. ^1^H NMR (500 MHz; CDCl_3_)/δ (ppm): 8.42 (s, 1H), 8.11 (d, *J* = 9.7 Hz, 1H), 7.54 (dd, *J* = 8.8, 3.4 Hz, 2H), 7.39 (dd, *J* = 8.6, 2.8 Hz, 1H), 7.32 (d, *J* = 2.5 Hz, 1H), 7.12 (dd, *J* = 8.9, 2.5 Hz, 1H), 7.06 (d, *J* = 9.7 Hz, 1H), 6.13 (dd, *J* = 14.6, 3.6 Hz, 1H), 5.25 (s, 2H), 3.95 (s, 3H). ^13^C NMR (125 MHz; DMSO)/δ (ppm): ^13^C NMR (126 MHz, CDCl_3_) δ 160.01, 159.16, 158.80, 156.92, 156.83, 156.44, 152.88, 151.18, 145.83, 142.17, 137.70, 134.31, 130.79, 130.04, 129.53, 123.08, 122.26, 115.89, 111.43, 104.61, 104.50, 104.37, 104.05, 103.92, 71.01, 70.79, 40.93. ^19^F NMR (470 MHz, CDCl_3_)/δ (ppm):–94.94 (dd, *J* = 63.2, 14.7 Hz),–113.31,-113.45; HRMS: Calculated for [M+H]^+^ (M = C_2__0_H_1__5_F_2_N_4_O_4_) = 413.1061, actual *m/z* = 413.1055.

### Radiochemistry

#### General Considerations

Unless otherwise stated, reagents and solvents were commercially available and used without further purification: sodium chloride, 0.9% USP, and sterile water for injection, USP, were purchased from Hospira; ethanol was purchased from American Regent; HPLC grade acetonitrile was purchased from Fisher Scientific. Other synthesis components were obtained as follows: sterile filters were obtained from Millipore; sterile product vials were purchased from Hollister-Stier; C18 Sep-Paks were purchased from Waters Corporation. C18 Sep-Paks were flushed with 10 mL of ethanol followed by 10 mL of water prior to use. Radio-HPLC was performed using a Shimadzu LC-2010A HT system equipped with a Bioscan B-FC-1000 radiation detector.

#### Procedure for Radiochemical Synthesis of [^18^F]1

[^18^F]Fluoride was prepared using an automated GE TRACERLab FX_FN_ synthesis module. The TRACERLab was configured as shown in Supplementary Figure 1 and the reagent vials were loaded as follows: Vial 1: potassium carbonate (3.5 mg in 0.5 mL water); Vial 2: kryptofix-2.2.2 (15 mg in 1.0 mL MeCN); Vial 3: precursor (4.0 mg in 950 μL DMSO and 5.0 μL sat. NH_4_Cl); Vial 6: HPLC buffer (35% acetonitrile, 20 mM NH_4_OAc, 0.2% acetic acid, 3.0 mL); Vial 7: 0.9% sodium chloride for injection, USP (4.18 mL); Vial 8: ethanol (0.66 mL) and Tween-80 (0.16 mL); and Vial 9: sterile water for injection, USP (10 mL); round bottom flask: water (50 mL); product vial: 0.9% sodium chloride for injection, USP (5.0 mL).

Fluorine-18 was produced *via* the ^18^O(p,n)^ 18^F nuclear reaction using a GE PET Trace cyclotron equipped with a high yield fluorine-18 target at 55 μA to produce 74 GBq (2 Ci) of fluorine-18. The [^18^F]Fluoride was delivered from the cyclotron (in a 2.5 ml bolus of [^18^O]H_2_O) and trapped on a QMA-Light Sep-Pak, which had been preconditioned with sodium bicarbonate, to remove [^18^O]H_2_O. [^18^F]Fluoride was then eluted into the reaction vessel using aqueous potassium carbonate (3.5 mg in 0.5 mL of water). A solution of kryptofix-2.2.2 (15 mg in 1.0 mL of acetonitrile) was then added to the reaction vessel and the [^18^F]fluoride was dried by azeotropic evaporation of the water-acetonitrile mixture. Evaporation was achieved by heating the reaction vessel to 100°C. The reactor was then cooled to 90°C, and precursor was added with stirring for 3 min. Subsequently, the reaction mixture was cooled to 50°C, followed by the addition of HPLC buffer (3.0 mL). The reaction was loaded onto a semipreparative column (Luna PFP, 250 × 10 mm, 35% Acetonitrile, 20 mM NH_4_OAc, 0.2% AcOH, flow rate = 4 mL/min). The product peak (∼82-86 min retention time, see Supplementary Figure 2 for a typical HPLC trace) was collected and diluted into a round-bottom flask containing 50 mL of water. The solution was then passed through a C-18 Sep-Pak to trap the product on the C-18 cartridge. The C18 cartridge was washed with 10 mL of sterile water. The product was eluted with 0.82 mL of ethanol/Tween 80 solution (0.66 mL of ethanol in 0.16 mL in Tween 80), followed by 9.5 mL of normal saline. The final formulation was passed through a 0.2 μm sterile filter into a sterile dose vial. The final product was obtained in 2446 ± 17.8 MBq (66.1 ± 17.7 mCi), 4.1% decay corrected yield,>98% RCP (see Supplementary Figure 3), pH = 5–5.5, *n* = 3 in 120 min from the end of bombardment. Identity was confirmed via co-injection with unlabeled reference standard (Supplementary Figure 4) and the product was stable for at least 150 min post-end-of-synthesis (Supplementary Figure 5).

### Preclinical Positron Emission Tomography Imaging

#### General Considerations

All animal studies were performed in accordance with the standards set by the University of Michigan Institutional Animal Care and Use Committee (IACUC).

#### Rodent Small Animal Positron Emission Tomography Imaging Protocol

PET imaging studies were performed for **[^18^F]1** in Sprague-Dawley female rats (*n* = 3, animal weights = 283–411 g) using a Concorde MicroPET P4 gantry (Knoxville, TN) scanner. The animals were anesthetized (isoflurane), placed on a nose cone and positioned in the scanner for imaging. Anesthesia was maintained throughout the entire study. Following a transmission scan, the animals were injected intravenously (*i.v*.) *via* tail vain catheter as a bolus over 1 min with **[^18^F]1** (447–476 μCi in 140–150 μL of saline) and the head was imaged for 120 min. In each case, emission data were corrected for attenuation and scatter and reconstructed using the 3D maximum *a priori* (3D MAP) method. By using a summed image, regions of interest were defined for the whole brain on multiple planes. The volumetric regions of interest were then applied to a full dynamic data set to generate time-radioactivity curves (TACs).

#### Non-human Primate Positron Emission Tomography Imaging Protocol

Imaging studies were performed on Microsystem (Knoxville, TN) R4 microPET in two intact, mature female rhesus monkeys (*n* = 2, animal weights 9.6–10.2 kg). The animals were anesthetized in the home cage with telazol and transported to the PET facility. Subjects were intubated for mechanical ventilation, and anesthesia was continued with isoflurane. Anesthesia was maintained throughout the duration of the PET scan. A venous catheter was inserted into one hind limb and the monkey was placed on the PET gantry with its head secured to prevent motion artifacts. Ten minutes later, 3.5–5.3 mCi of **[^18^F]1** was administered in a bolus dose over 1 min, and the brain imaged for 120 min (5 × 2 min frames – 4 × 5 min frames – 9 × 10 min frames). Emission data were collected beginning with the injection, and continued for 120 min. Collection of vitals (HR, SPO_2_, EtCO_2_, and respiratory rate) was carried out during the whole scan. Data were corrected for attenuation and scatter and reconstructed using the three- dimensional–maximum *a priori* method (3D MAP algorithm). By using a summed image, regions of interest were defined for the whole brain and different brain regions on multiple planes. The volumetric regions of interest were then applied to a full dynamic data set to generate TACs.

### Autoradiography

Frozen blocks (1×1 inch) of postmortem brain tissue samples from HD patients and a normal control (age range from 49 to 85) were used for the autoradiography binding studies (Table 1). Tissue was obtained from the University of Michigan Alzheimer’s Disease Center Brain Bank. Frozen blocks were sliced into 20 μm sections using a Hacker Instruments cryostat set to –15°C. Tissue was thaw-mounted on the 1×3 inch polylysine-subbed glass slides. Sections used for autoradiography experiments were pre-conditioned for 5 min with phosphate buffer saline (PBS) pH 7.4 at 25°C, and incubated in varying concentrations of **[^18^F]1** (0.05–5 nM) for 30 min at room temperature. Nonspecific binding (NSB) was determined by coincubation in the presence of 10 μM unlabeled **1F** (dissolved in 1 mL methanol). Sections were washed twice (1 min each) in PBS followed by a distilled water rinse, all at 4°C. Sections were dried under a stream of air and opposed to a phosphoimager screen for 10 min. Aliquots of the stock solutions were placed on a TLC plate and co-exposed with tissue sections as a standard curve. After development (GE/Fuji Typhoon FLA 7,000), image densitometry was analyzed with ImageQuant software (Fuji). Phosphoimager units were converted to femtomoles on the basis of image densities overlying the standards and the specific activity of the radioligand. Data was analyzed with Excel and graphs made with SigmaPlot.

**TABLE 1:** Brain tissues samples used in pre-clinical evaluation.

**Patient #**	**Tissue samples**	**Age**	**Sex**	**Postmortem delay (h)**
1604 (HD)	Caudate, putamen, cortex	56	M	4.0
1603 (HD)	Putamen, cortex	49	M	8.5
1607 (HD)	Putamen	61	F	5.5
1617 (CON)	Putamen	85	F	21.0

### Immunohistochemistry

#### Tissue Fixing

Brain tissue sections were removed from storage at –80°C and thawed for 5 min before incubating in Davidson’s fixative (8.1% formaldehyde, 33.3% ethanol, 11.1% acetic acid, Eosin Y stain) for 24 h at room temp. Sections were then quickly rinsed in 70% ethanol to remove residual formaldehyde. All incubations were carried out at room temperature.

#### Primary Antibody Staining

Fixed tissue sections were incubated in PBS with 1% SDS for 5 min. Sections were then washed 3 × 5 min in PBS before quenching in 70% methanol with 0.3% hydrogen peroxide for 15 min. All slides were washed 3 × 5 min in PBS-T (PBS, 0.4% Triton-X-100, pH 7.4) and blocked for 30 min with PBS-TBA (PBS, 0.4% Triton-X-100, 1% BSA, 0.025% sodium azide, pH 7.4) before incubating in a 1:1000 dilution of primary antibody (anti-huntingtin ABN903; Sigma Aldrich) in PBS-TBA overnight. Finally, brain sections were washed 3 × 5 min in PBS-T to remove unbound antibody. All incubations were carried out at room temperature.

#### Secondary Antibody Staining

Tissue sections were washed 3 × 5 min in PBS-T and incubated in a 1:200 dilution of secondary antibody (anti-goat-IgG, Vector Laboratories BA-5,000, anti-rabbit-IgG, Vector Laboratories BA-1000) in PBS-TBA for 2 h and washed 3 × 5 min with PBS-T. All incubations were carried out at room temperature.

#### Visualization

Slides were developed as instructed using the VECTASTAIN Elite ABC Kit (Standard) (Vector Laboratories PK-6100). Tissue sections were then washed 3 × 5 min in PBS-T before incubating for 4 min in a 0.5% w/v solution of diaminobenzidine in PBS-T (filtered) with 0.001% hydrogen peroxide and finally counterstaining with Giemsa stain. All incubations were carried out at room temperature.

## Results and Discussion

### Development of a Radiosynthesis of [^18^F]1

**SCHEME 1:**
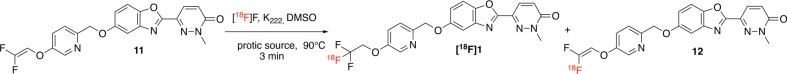
Synthesis of cold standard **1** and precursor **11** for radiosynthesis of **[^18^F]1**.

The synthesis of required gem-difluoroalkene precursor **11** was envisioned as shown in Scheme 1, as an additional step of the route to the required standard. The selective protection of phenolic group of 6-(hydroxymethyl)pyridin-3-ol **2** was carried out with 2,2,2-trifluoroethylmethanesulfonate yielding trifluoroethyl protected intermediate **3** in 41% yield. Analog **3** was reacted with 2-methyl-1,3-benzoxazol-5-ol and Tsunoda reagent (cyanomethylenetributylphosphorane) to construct the benzoxazole intermediate **5** in 73% yield. Next, ring opening of benzoxazole intermediate **5** was achieved by treatment with 2M HCl in ethanol which yielded amino alcohol **7** in quantitative yield.

The amino alcohol **7** was subjected to peptide coupling with 1-methyl-6-oxo-1,6-dihydropyridazine-3-carboxylic acid using EDCI/pyridine to give amide product **9** in 54% yield. The cyclization of amide **6** to synthesize reference standard **1** was carried out using at reflux in toluene using a Dean-Stark trap and *p*-toluene sulfonic acid as an acid catalyst in an 80% yield. After successful synthesis of standard **1**, the next step was to construct gem-difluoro enol ether precursor **11** for its application to try radiochemistry. We attempted generation of the precursor by subjecting **1** with *n*-BuLi at –78°C, which has been reported for other PET imaging agent precursors (Fawaz et al., 2014), but the conditions did not result in the desired product and opened pyridazine ring of **1** was instead observed.

To synthesize difluoro enol ether precursor **11**, we thought to install the olefin in the initial stages of the synthetic route. As the opening of the pyridazine ring had been the issue, subjecting the simpler intermediate of pyridine **3** to *n*-BuLi for HF elimination reaction was a promising alternate route to the required precursor, **11**. So, analog **3** was subjected to 3 equivalents of *n*-BuLi conditions at –78°C for 45 min as per literature reports (Fawaz et al., 2014). Gratifyingly, we were able to isolate (5-((2,2-difluorovinyl)oxy)pyridin-2-yl)methanol intermediate **4** in 19% yield (Scheme 1). Following the route described previously for standard **1**, (5-((2,2-difluorovinyl)oxy)pyridin-2-yl)methanol intermediate **4** was treated with 2-methyl-1,3-benzoxazol-5-ol and Tsunoda reagent (cyanomethylenetributylphosphorane) to obtain benzoxazole intermediate **6** in 54% yield.

The ring opening of benzoxazole intermediate **6** yielded aminoalcohol intermediate **8** in quantitative yield. Further, coupling of 2-amino-4-((5-((2,2-difluorovinyl)oxy)pyridin-2-yl)methoxy)phenol **8** with 1-methyl-6-oxo-1,6-dihydropyridazine-3-carboxylic acid in EDCI/pyridine yielded amide **10** in 26% yield. Next, cyclization of amide **10** using the acid catalyzed toluene reflux with a Dean-Stark trap yielded gem-difluoroalkene precursor **11** in 74% yield (Scheme 1).

To accomplish the radiolabeling to generate **[^18^F]1**, previously reported radiochemical conditions for this chemistry developed by the Riss group and used for [^18^F]lansoprazole from our group were evaluated (Fawaz et al., 2014). Gem-difluoroalkene precursor **11** (2.3 mg) was dissolved in a mixture of anhydrous dimethyl sulfoxide (500 μL) and isopropanol (36 μL) and reacted with azeotropically dried [^18^F]fluoride at 90°C for 3 min (Entry 1). When subjected to these conditions, we obtained the desired product **[^18^F]1** in only 0.01% radiochemical yield (RCY) and in 1:10 ratio of trifluoromethyl (**[^18^F]1**)/difluoro-alkene (**12**) (Table 2). While laying the foundation of this chemistry, Pike and co-workers (Aigbirhio et al., 1993) explained that anhydrous conditions favored the generation of radiolabeled alkene *via* an elimination-addition mechanism. Further Riss et al. (2012) explained that protic additives enhanced the ratio of trifluoromethyl derivatives over the alkene (Riss and Aigbirhio, 2011; Riss et al., 2012), owing to the quenching of the intermediate anion before eliminating a fluoride anion (Landini et al., 1989). Next, we tried other protic additives (e.g., ammonium chloride, triflate or carbonate) that have been previously utilized for production of [^18^F]*N*-methyl-lansoprazole (NML) for clinical use (Fawaz et al., 2014; Kramer et al., 2020). The optimal reaction solvent consisted of a mixture of anhydrous DMSO (950 μL) and saturated ammonium chloride (5.0 μL), as optimized for NML (Fawaz et al., 2014). Gratifyingly, the reaction resulted in product formation in a 4–5% (*n* = 3) radiochemical yield, and a satisfactory ratio of [^18^F]trifluoromethyl **[^18^F]1/**[^18^F]gem-difluoroalkene **12** (1:3).

**TABLE 2:** Conditions for the radiosynthesis of **[^18^F]1**.

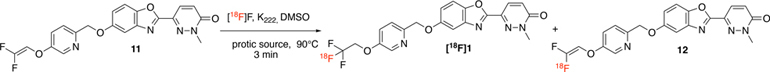			

**S. No.**	**Reaction conditions**	**Combined RCY of [^18^F]1 and 12**	**Ratio of [^18^F]1/12**
1.	500 μL of DMSO and 36 μL of IPA^a^	0.01% (*n* = 2)	1:10
2.	950 μL of DMSO and 5 μL of sat. NH_4_Cl^b^	4–5% (*n* = 6)	3:1

*^a^Precursor (2.3 mg). IPA, isopropyl alcohol.*

*^b^Precursor (4.0 mg).*

Upon establishing the radiochemistry conditions, we turned our attention to the development of a suitable HPLC method for purification of **[^18^F]1**, which we expected to be challenging given the structural similarities of **[^18^F]1** and **12**. Reflecting this, traditional reverse-HPLC stationary phases like C18 failed to achieve reasonable separation between the two compounds. Fortunately, perfluorophenyl-capped matrix [Luna-PFP(2), Phenomenex] as the stationary phase worked well in both the semipreparative and analytical separations (see representative HPLC traces in the Supporting Information). Purification of **[^18^F]1** was achieved using semipreparative HPLC conditions (see Supplementary Figure 2 for a typical HPLC trace), which enabled separation of the desired product **[^18^F]1** (t_R_ = 82–86 min) and [^18^F]gem-difluoroalkene **12** (t_R_ = 73–80 min). While retention times are somewhat long, and there is likely further scope for optimization, this method provided adequate separation of **[^18^F]1** and **12** for this preliminary study. For reformulation, the purified **[^18^F]1** was trapped on a C18 (Waters, 1cc vac) cartridge, the cartridge was rinsed with water to remove the residual HPLC solvent/buffers and eluted with ethanol (0.5 mL) and saline (9.5 mL) for injection. During the sterile filtration step, we noticed that the dose was retained on the filter membrane, losing 40% of the imaging agent on the filter. In order to avoid this loss of dose, we screened different filters (see Supplementary Material) and conditions. Gratifyingly, we were able to successfully reformulate the dose in a mixture of ethanol (660 μL), Tween-80 (160 μL), and saline (9.5 mL), which facilitated sterile filtration without loss of dose. The formulation had the added advantage that the dose was also no longer retained on the syringes utilized for *i.v.* injection during preclinical imaging studies. Full automated synthesis provided **[^18^F]1** in 4–5% yield (2,446 ± 17.8 MBq, 66.1 ± 17.7 mCi, *n* = 3, decay corrected radiochemical yield, based upon 2.0 Ci of [^18^F]fluoride),>98% RCP and molar activities = 16.5 ± 12.5 GBq/μmol (445 ± 339 Ci/mmol). The radiochemical purity of formulated **[^18^F]1** was analyzed with radio-HPLC to determine stability of a dose kept at room temperature for 2.5 h post-end-of-synthesis; **[^18^F]1** did not show any evidence of decomposition and RCP remained>95% (see Supplementary Figure 6).

### Preclinical Evaluation of [^18^F]1

#### *In vitro* Autoradiography and Immunohistochemistry

**FIGURE 3:**
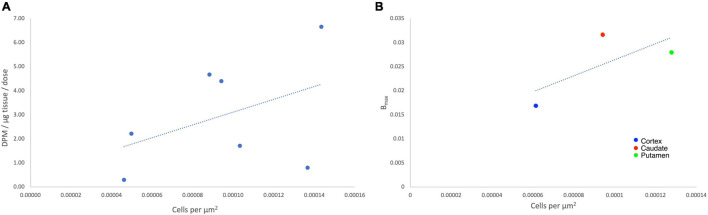
Autoradiography correlates with immunohistochemistry for individual data points **(A)** and average B_*max*_ (fmol/μg) values **(B)**.

To determine the suitability of **[^18^F]1** for *in vivo* experiments, we first undertook an *in vitro* autoradiography using post-mortem brain tissue samples from HD patients as well as a control subject (Table 1 and Supplementary Figures 7–11). *In vitro* binding experiments were used to measure **[^18^F]1** affinity to *m*HTT aggregates in the HD brain sections, and the K_d_ of **[^18^F]1** for *m*HTT was 2.30 nM. A series of saturation binding experiments using postmortem HD brain slices was next performed (Table 3). Results of saturation binding of this radiotracer in postmortem HD brain slices suggested that the binding in caudate and putamen is specific and saturable (Table 3, entries 1 and 2), consistent with HD being a disease known to predominantly impact neurons in the basal ganglia, particularly in the earliest stages (Reiner et al., 2011; Tang and Feigin, 2012). Scatchard analysis suggested that the binding fitted to a single binding site (see Supplementary Material). As HD progresses there is also involvement of the cerebral cortex and other subcortical structures (Burgold et al., 2019). We observed saturable binding of **[^18^F]1** in HD cortical tissue samples (Table 3, Entry 3), but it was substantially lower that the caudate and putamen sections. Lastly, as expected, we also observed no evidence of saturable binding of **[^18^F]1** in a control putamen sample (Table 3, Entry 4). Non-specific binding (identified by co-incubation with 10 μM **1F**) was observed in the white matter. To validate the autoradiography data, we conducted immunohistochemistry with anti-huntingtin antibody (ABN903) to identify *m*HTT aggregates in adjacent brain sections. Considering individual brain samples revealed a weak correlation (*R* = 0.433) between *m*HTT positive cells per μm^2^ and disintegrations per μg of tissue/decay corrected dose (Figure 3A). This trend might only be weakly discernible given heterogenous distribution of *m*HTT aggregates and the small sample size available from our brain brank. Indeed, a stronger correlation (*R* = 0.714) was apparent when considering the trend between *m*HTT positive cells per μm^2^ identified by IHC and averaged across samples of a given brain region, and the calculated B_*max*_ from the binding studies for the same brain region (Figure 3B).

**TABLE 3:** Preclinical evaluation of **[^18^F]1**.

**Entry**	**Brain sample**	**Saturable Binding**	**B_*max*_ (fmol/μg)**	**B_*max*_ (nM)**	**BP (B_*max*_/K_*d*_)**	**Cells (per μm^2^)**	**n**
1	HD (Putamen)	+	0.0280 ± 0.008	28.0 ± 8	12.2	0.000128 ± 0.0000215	3
2	HD (Caudate)	+	0.0317 ± 0.0184	31.7 ± 18.4	13.8	0.0000940	1
3	HD (Cortex)	+/-	0.0169 ± 0.0164	16.9 ± 16.4	7.4	0.0000613 ± 0.0000234	3
4	CON (Putamen)	–	N/A	N/A	N/A	N/A	1

#### *In vivo* Positron Emission Tomography Imaging

**FIGURE 4:**
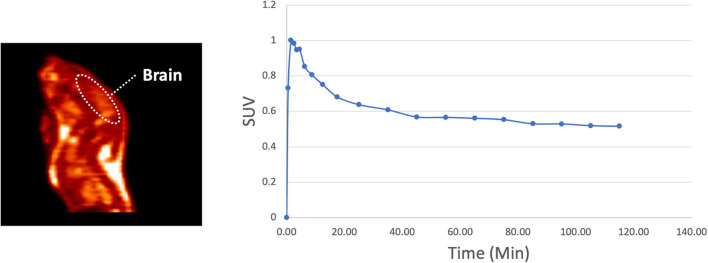
Representative rodent PET imaging with **[^18^F]1**: summed sagittal image (0–120 min post *i.v.* injection of the radioligand) and average whole brain time-radioactivity curve (*n* = 3).

The *in vivo* behavior of **[^18^F]1** was initially investigated in rodents. PET imaging studies were performed with **[^18^F]1** in female Sprague-Dawley rats (*n* = 3). A region-of-interest (ROI) was defined for the whole brain in the reconstructed PET data, and the summed data was used to generate a whole brain TAC (Figure 4). The data was converted to standardized uptake values (SUVs) and plotted for the 120 min dynamic imaging window. PET scans of the 3 rats revealed rapid uptake of **[^18^F]1** in the brain, with peak uptake occurring in 90 s (∼1,750 nCi/cc, corresponding to SUV_*max*_ of ∼1.0) and subsequent washout throughout the duration of the scan. We observed about 30% clearance in 30 min, and about 45% clearance in 40 min.

**FIGURE 5:**
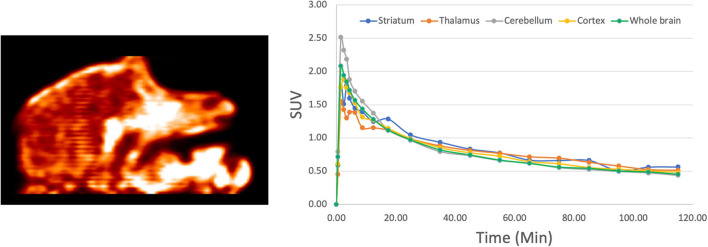
Representative NHP PET imaging with **[^18^F]1**: summed sagittal image (0–120 min post *i.v.* injection of the radioligand) and average regional time-radioactivity curves (*n* = 2).

These encouraging results prompted us to next examine the *in vivo* imaging properties of **[^18^F]1** in non-human primates (NHPs). PET imaging studies were performed for **[^18^F]1** in mature female rhesus monkeys (*n* = 2). ROIs were drawn for the whole brain, as well as numerous brain regions (cortex, cerebellum, thalamus, striatum), and the summed data was analyzed to generate regional TACs. The data was converted to SUV and plotted for the 120 min imaging window (Figure 5). Results were analogous to rodent scans, with high brain uptake of **[^18^F]1** apparent in both monkey scans and peak uptake occurring in ∼90 s (∼1,000 nCi/cc, corresponding to SUV_*max*_ of ∼2.0). Following peak uptake, **[^18^F]1** washed out from the brain. The time-radioactivity curves revealed about 30–40% clearance in 20 min, and about 50–60% clearance in 40 min. The results confirmed that the scaffold is blood-brain barrier (BBB) permeable and demonstrated quick wash-out from the brain, and low background/non-specific signal.

After an intravenous injection, **[^18^F]1** penetrated the intact blood–brain barrier (BBB) of both rodents and NHPs efficiently, and peak uptake occurred in ∼90 s. Because there were no mutant HTT aggregates in the healthy monkey or rodent brain, as expected, **[^18^F]1** did not display any specific binding or prolonged retention in the brain. The performance is comparable with literature data for [^11^C]CHDI-180R [e.g., SUV 2.7–3.0 in NHP imaging studies (Liu et al., 2020)].

## Conclusion

In summary, an automated radiosynthesis of an ^18^F PET imaging agent for *m*HTT has been developed for imaging patients with Huntington’s disease. Highlights of the current method are its straightforward chemistry, simplicity, good radiochemical yields, and adaption to a commercial radiochemistry synthesis module for automated production of the radioligand in high purity. Imaging studies exhibited good brain uptake in rats and non-human primates, and autoradiography studies with post-mortem human HD brain tissue studies showed specific binding and evidence of correlation with *m*HTT protein aggregates identified by immunohistochemistry. Overall **[^18^F]1** is a promising candidate for imaging *m*HTT with PET to support disease management, track disease progression and evaluate experimental HD therapies. Future studies aimed at clinical translation of **[^18^F]1** will determine the safety profile of the radiotracer (pharmacology/toxicology and dosimetry studies), establish the metabolism, validate a synthesis to provide tracer suitable for clinical use and further validate the specificity of the signal for *m*HTT aggregates.

## Data Availability Statement

The original contributions presented in the study are included in the article/Supplementary Material, further inquiries can be directed to the corresponding author/s.

## Ethics Statement

The animal study was reviewed and approved by the University of Michigan IACUC.

## Author Contributions

PS designed the research. TK, AB, AL, TD, JS, JA, and WW performed the research. TK and AB contributed new reagents and analytical tools. TK, AB, AL, WW, TD, and PS analyzed the data. TK, AB, and PS wrote the manuscript. All authors reviewed the manuscript.

## Conflict of Interest

The authors declare that the research was conduscted in the absence of any commercial or financial relationships that could be construed as a potential conflict of interest.

## Publisher’s Note

All claims expressed in this article are solely those of the authors and do not necessarily represent those of their affiliated organizations, or those of the publisher, the editors and the reviewers. Any product that may be evaluated in this article, or claim that may be made by its manufacturer, is not guaranteed or endorsed by the publisher.
