# Supplementary material for: Synthesis and Evaluation of a Fluorine-18 Radioligand for Imaging Huntingtin Aggregates by Positron Emission Tomographic Imaging
Source: Front Neurosci. 2021 Dec 2;15:766176. doi: 10.3389/fnins.2021.766176 (PMC8675899; doi:10.3389/fnins.2021.766176)
Supplement: Supplementary file 1 [file Data_Sheet_1.pdf]

Supplementary Material

Synthesis and Evaluation of a Fluorine-18 Radioligand for Imaging Huntingtin Aggregates by Positron Emission Tomographic Imaging

Table of Contents

1. Radiochemistry……………………………………..…………………………………………….….S2

2. Preclinical Experiments…… ..………………………………………………………….…...………........S7
   2.1 Saturation binding curves ...…………………………………………………….....………........S7
   2.2 Representative autoradiography images…………………………………………………………S9

3. ^{1}H NMR, ^{13}C NMR and HRMS Spectra……………………..………………………………………...S10
   6.1. (5-(2,2,2-Trifluoroethoxy)pyridin-2-yl)methanol (**3**)
   6.2. 2-Methyl-5-((5-(2,2,2-trifluoroethoxy)pyridin-2-yl)methoxy)benzo[*d*]oxazole (**5**)
   6.3. 2-Amino-4-((5-(2,2,2-trifluoroethoxy)pyridin-2-yl)methoxy)phenol (**7**)
   6.4. *N*-(2-Hydroxy-5-((5-(2,2,2-trifluoroethoxy)pyridin-2-yl)methoxy)phenyl)-1-methyl-6-oxo-1,6-di hydropyridazine-3-carboxamide (**9**)
   6.5. 2-Methyl-6-(5-((5-(2,2,2-trifluoroethoxy)pyridin-2-yl)methoxy)benzo[d]oxazol-2-yl)pyridazine 3(2*H*)-one (**1**)
   6.6. (5-((2,2-Difluorovinyl)oxy)pyridin-2-yl)methanol (**4**)
   6.7. 5-((5-((2,2-Difluorovinyl)oxy)pyridin-2-yl)methoxy)-2-methylbenzo[*d*]oxazole (**6**)
   6.8. 2-Amino-4-((5-((2,2-difluorovinyl)oxy)pyridin-2-yl)methoxy)phenol (**8**)
   6.9. *N*-(5-((5-((2,2-Difluorovinyl)oxy)pyridin-2-yl)methoxy)-2-hydroxyphenyl)-1-methyl-6-oxo-1,6-dihydropyridazine-3-carboxamide (**10**)
   6.10. 6-(5-((5-((2,2-Difluorovinyl)oxy)pyridin-2-yl)methoxy)benzo[*d*]oxazol-2-yl)-2-methylpyridazin-3(2*H*)-one (**11**)

1. Radiochemistry

6-(5-((5-(2,2-Difluoro-2-(fluoro-^{18}*F*)ethoxy)pyridin-2-yl)methoxy)benzo[*d*]oxazol-2-yl)-2-methyl pyridazin-3(2*H*)-one [^{18}F]1:

[^{18}F]**1** was synthesized as described in the main manuscript using a TRACERLab FX_{FN} configured as shown in Supplemental Figure 1. Purification *via* semi-preparative HPLC utilized 35% Acetonitrile, 20mM NH_4OAc, 0.2% Acetic acid mobile phase at a flow rate of 4 mL/min (for a representative tracer, see Supplemental Figure 2).

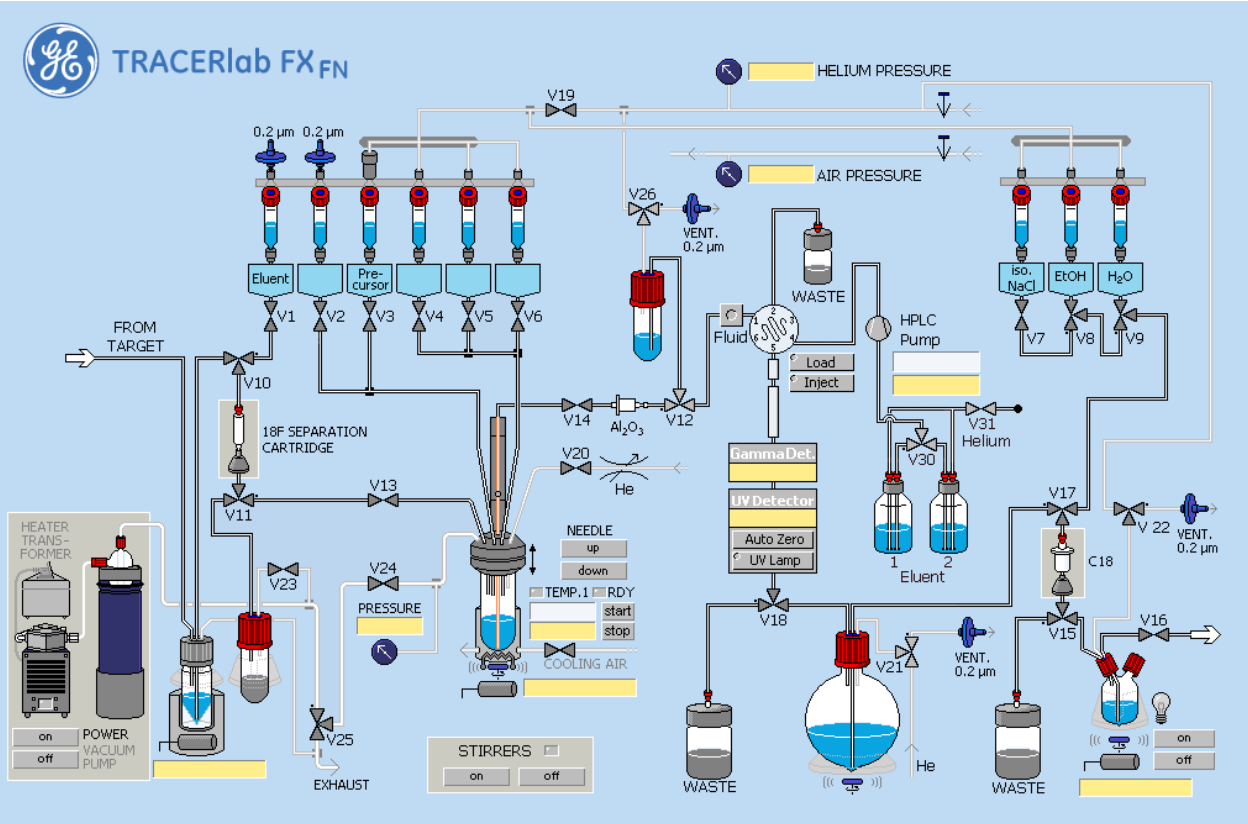

Supplement Figure 1: Synthesis module configuration.

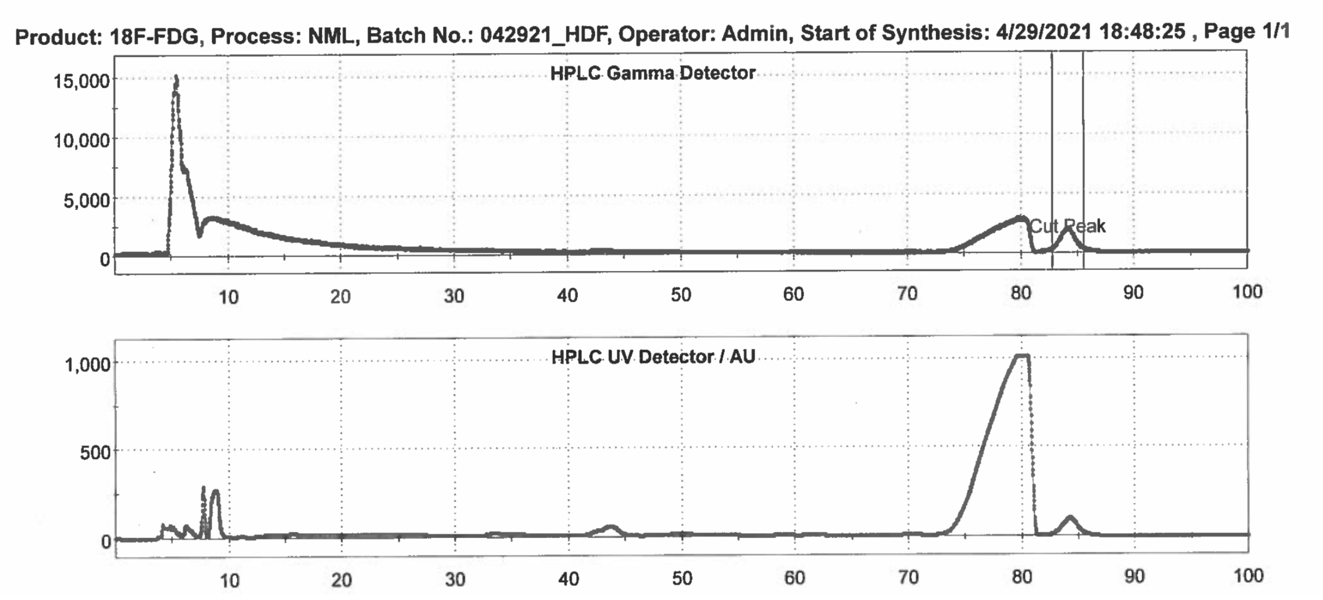

Supplemental Figure 2: Typical semi-preparative HPLC trace for **[^{18}F]1** (Column: Luna PFP, 250x10 mm-10μ, flow rate: 4 mL/min, mobile phase: 40% Acetonitrile, 20 mM NH_4OAc, 0.2% Acetic acid, retention time: ~81-85 min).

The decay corrected product yield was 66.1 ± 17.7 mCi (RCY 4.1%; n = 3). Analysis by radio-HPLC confirmed >98% RCP (Supplemental Figure 3), radiochemical identity via co-injection with unlabeled reference standard (Supplemental Figure 4) and that the product was stable for at least 150 min post-end-of-synthesis (Supplemental Figure 5).

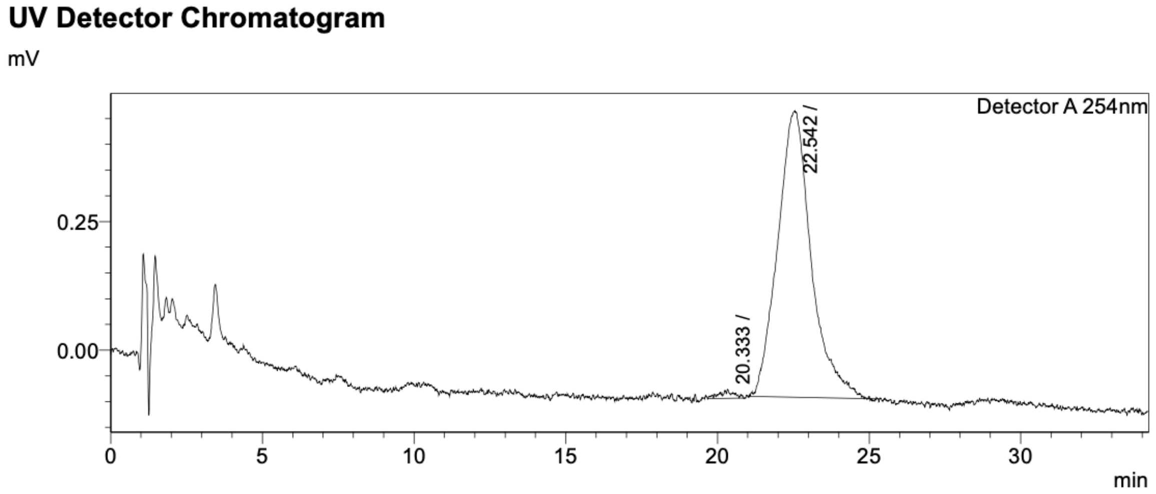

UV Detector

Detector A 254nm

Ret. Time	Area	Area%	Peak#
20.333	509	1.177	1
22.542	42719	98.823	2

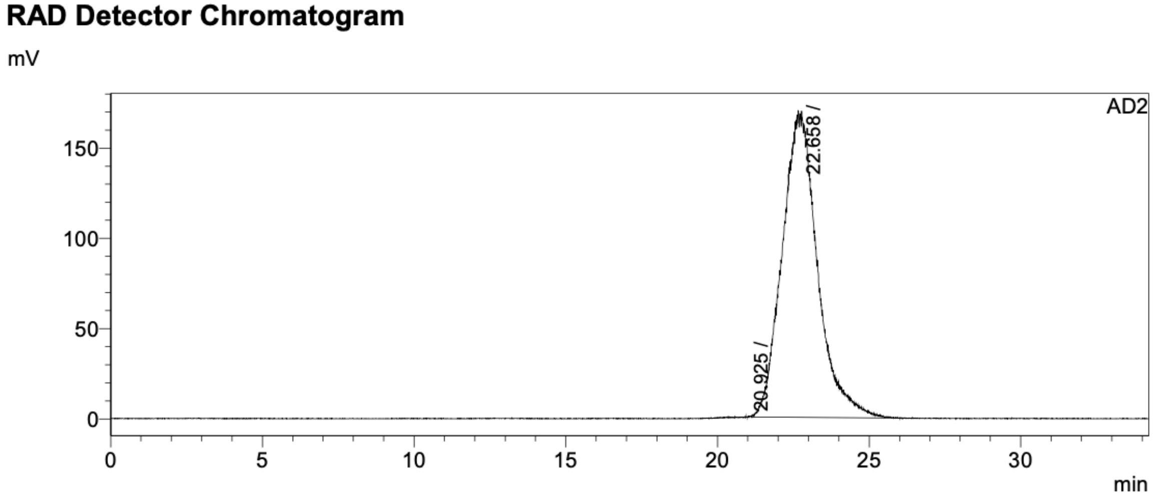

RAD Detector

AD2

Ret. Time	Area	Area%	Peak#
20.925	928	0.007	1
22.658	13628989	99.993	2

Supplemental Figure 3: Analytical HPLC trace for **[^{18}F]1**: Column: Luna PFP, 150x4.6 mm-5µ, flow rate: 2 mL/min, mobile phase: 30% Acetonitrile, 20 mM NH_4OAc, 0.2% Acetic acid, retention time: ~22-23 min.

UV Detector Chromatogram

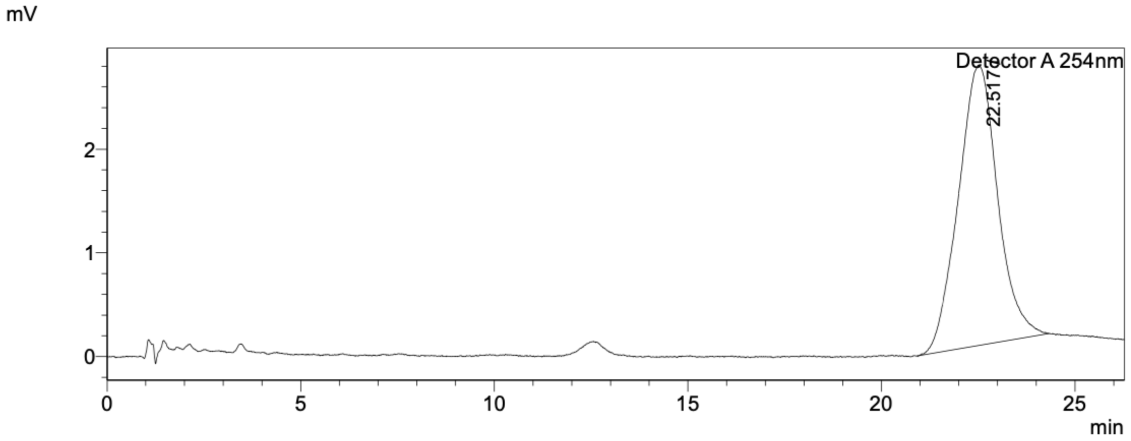

UV Detector

Detector A 254nm

Ret. Time	Area	Area%	Peak#
22.517	190884	100.000	1

RAD Detector Chromatogram

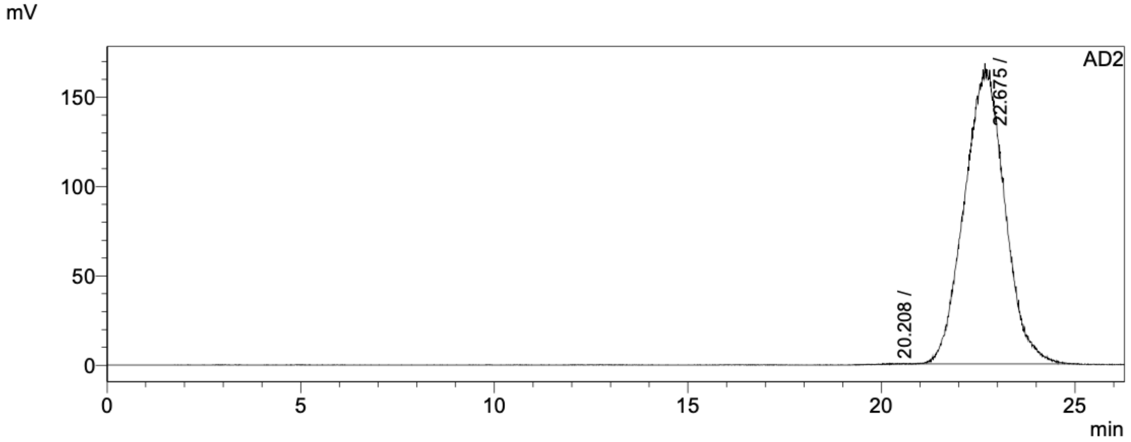

RAD Detector

AD2

Ret. Time	Area	Area%	Peak#
20.208	4075	0.034	1
22.675	12039721	99.966	2

Supplemental Figure 4: Analytical HPLC trace for **[^{18}F]1**: Coinjection of standard **1** compound (UV) with purified reaction product from radiochemical synthesis. Purified radiochemical product injected with cold standard, HPLC method repeated from Supp. Fig. 4. Integrations provided.

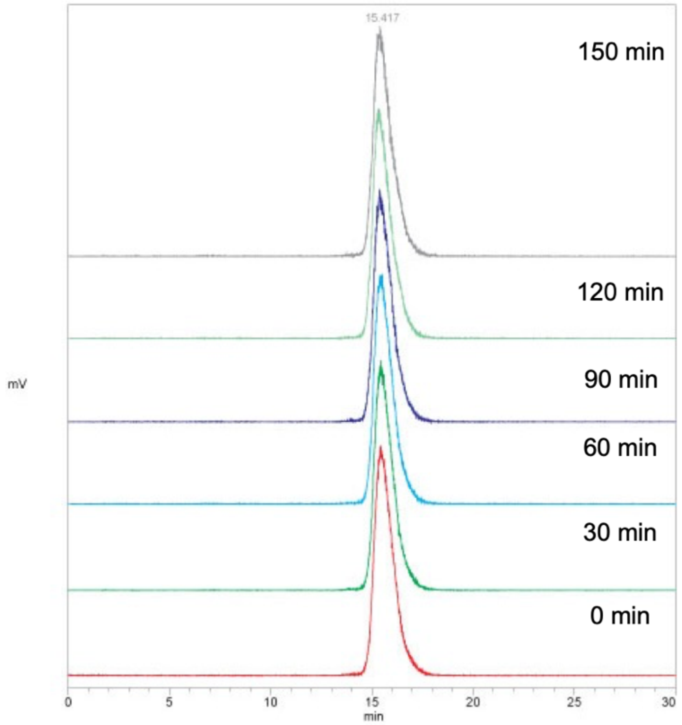

Time post EOS (min)	RCP (%)
0	99
30	99
60	99
90	99
120	99
150	99

Supplemental Figure 5: HPLC stability trace for **[^{18}F]1**: An Analytical HPLC trace of **[^{18}F]1** at different time points. Product is stable in solution for 150 minutes from the end of synthesis.

Screening of Different Filters

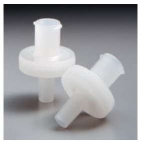

A

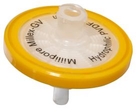

B

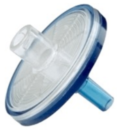

C

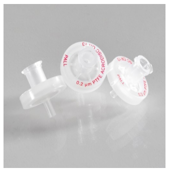

D

Entry	Filter type	Residue on filter (mCi)	Recovered in vial (mCi)	% Recovered
**A.**	Small GV (white)	4.01	2.25	40.3
**B.**	Large GV (yellow)	5.84	0.5	7.9
**C.**	GS filter (blue)	5.49	0.05	0.9
**D.**	PTFE (white-red)	2.05	0.07	3.3

Supplemental Figure 6: Screening of different filter types Previous reports of related molecules[1] used a formulation of 5% ethanol in saline. We observed with this formulation a loss of product to the filter and on the syringe, barrels used to transfer the dose for analysis. Alternate plastic types were evaluated for the sterile filter, but all showed retention of the **[^{18}F]1** and a loss of product. As a result, a formulation with Tween-80 was employed to improve the collection of **[^{18}F]1** in the sterile dose vial.

2. Preclinical Experiments

2.1 Saturation Binding Curves

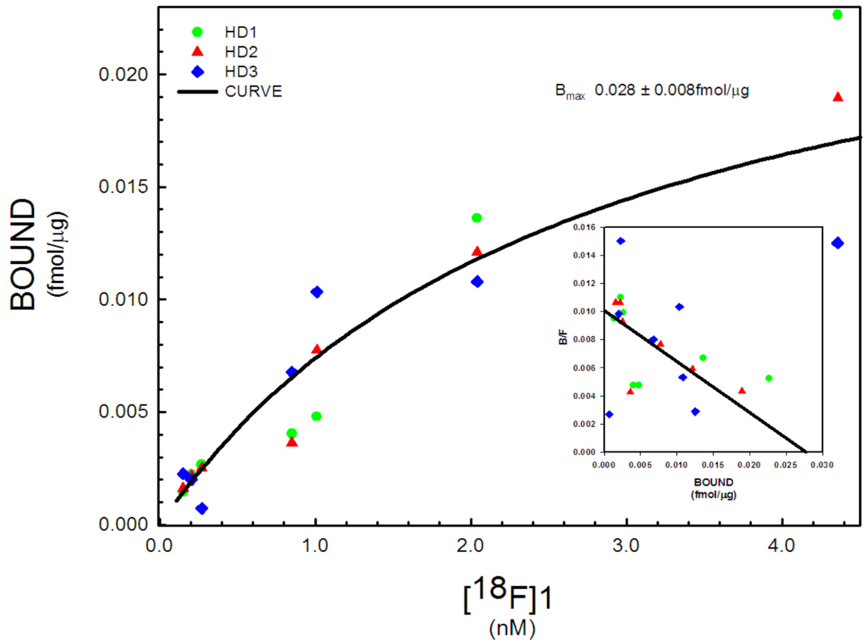

Supplementary Figure 7. Saturation analysis of **[^{18}F]1** showing saturable binding in HD patient putamen (n=3). Inset: Scatchard (Rosenthall) plot of specific binding.

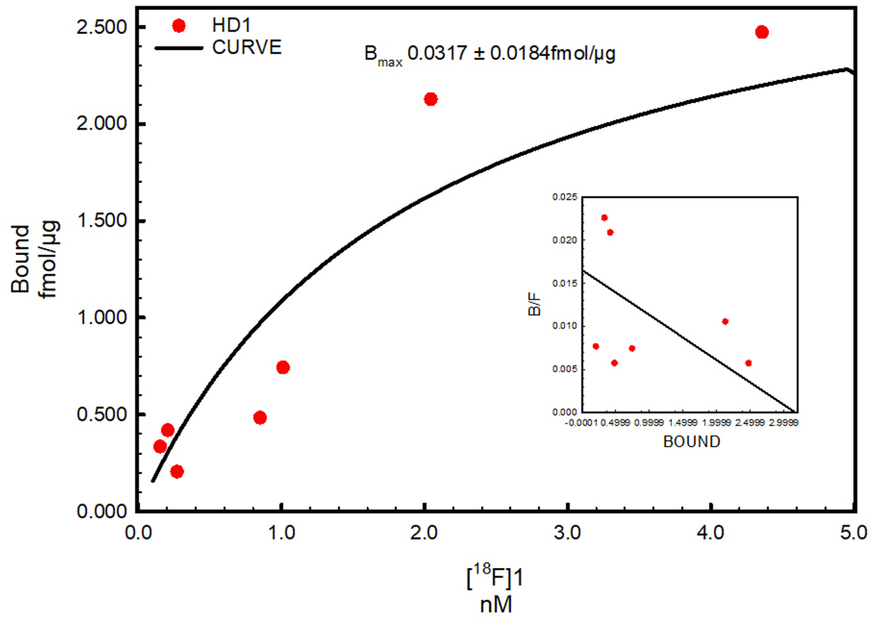

Supplementary Figure 8. Saturation analysis of **[^{18}F]1** showing saturable binding in HD patient caudate (n=1). Inset: Scatchard (Rosenthall) plot of specific binding.

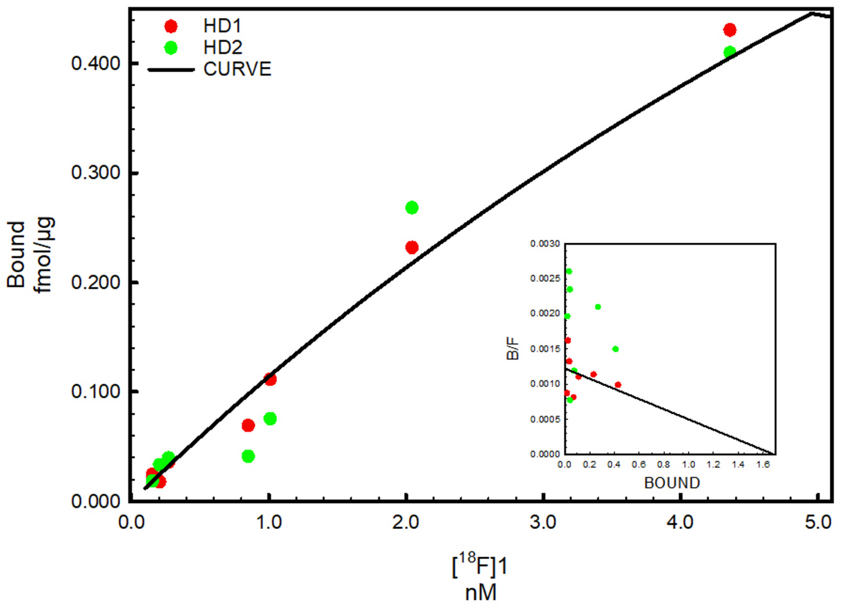

Supplementary Figure 9. Saturation analysis of **[^{18}F]1** showing saturable binding in HD patient cortex (n=1). Inset: Scatchard (Rosenthall) plot showing specific binding.

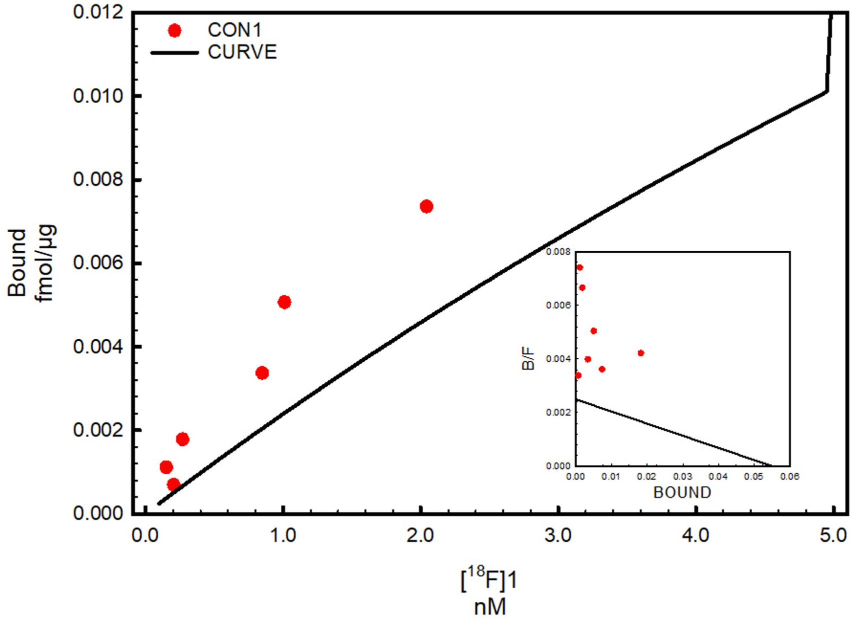

Supplementary Figure 10. Saturation analysis of **[^{18}F]1** showing no saturable binding in control subject putamen (n=1). Inset: Scatchard (Rosenthall) plot showing no specific binding.

2.2 Representative Autoradiography Images

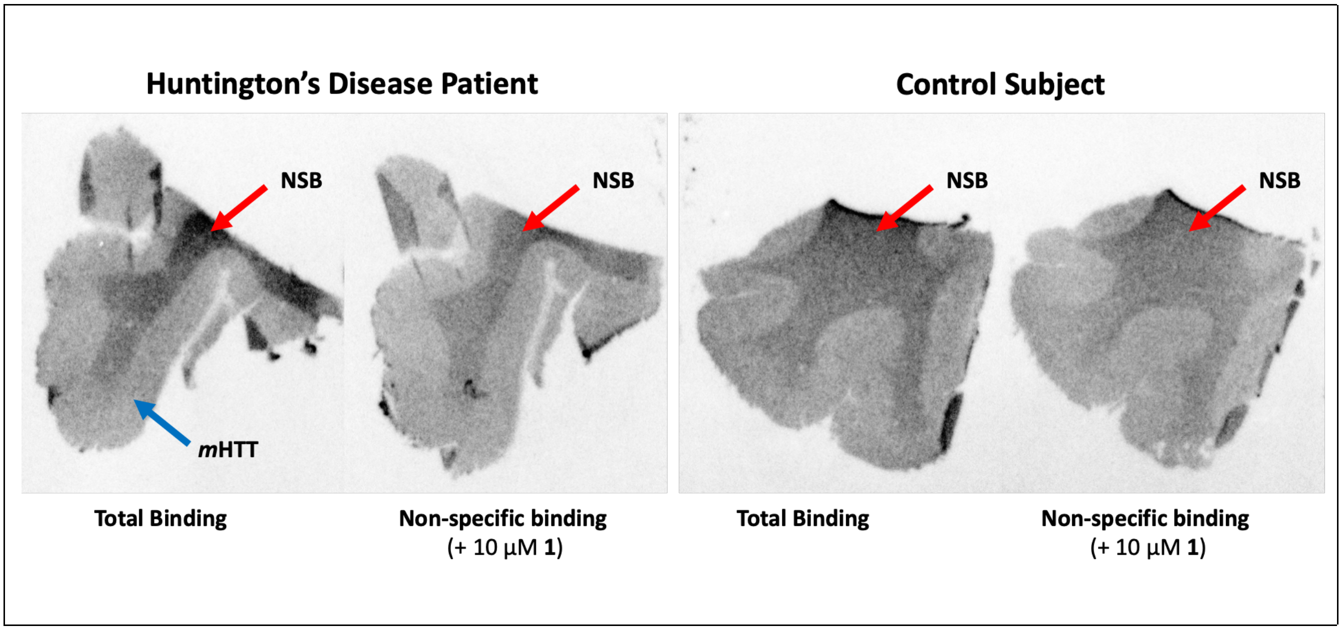

Supplementary Figure 11. Representative autoradiography images obtained using **[^{18}F]1** and cortical slices from an HD patient (**A**) and a control subject (**B**) showing total binding and non-specific (NSB) binding in the presence of 10 µM **1**. Displaceable binding to *m*HTT (**blue** arrow) is apparent in the HD patient but not the control, while non-specific binding in the white matter (**red** arrow) is apparent in both cases.

3. ^{1}H NMR, ^{13}C NMR and HRMS Spectra

3.1.(5-(2,2,2-Trifluoroethoxy)pyridin-2-yl)methanol (3):

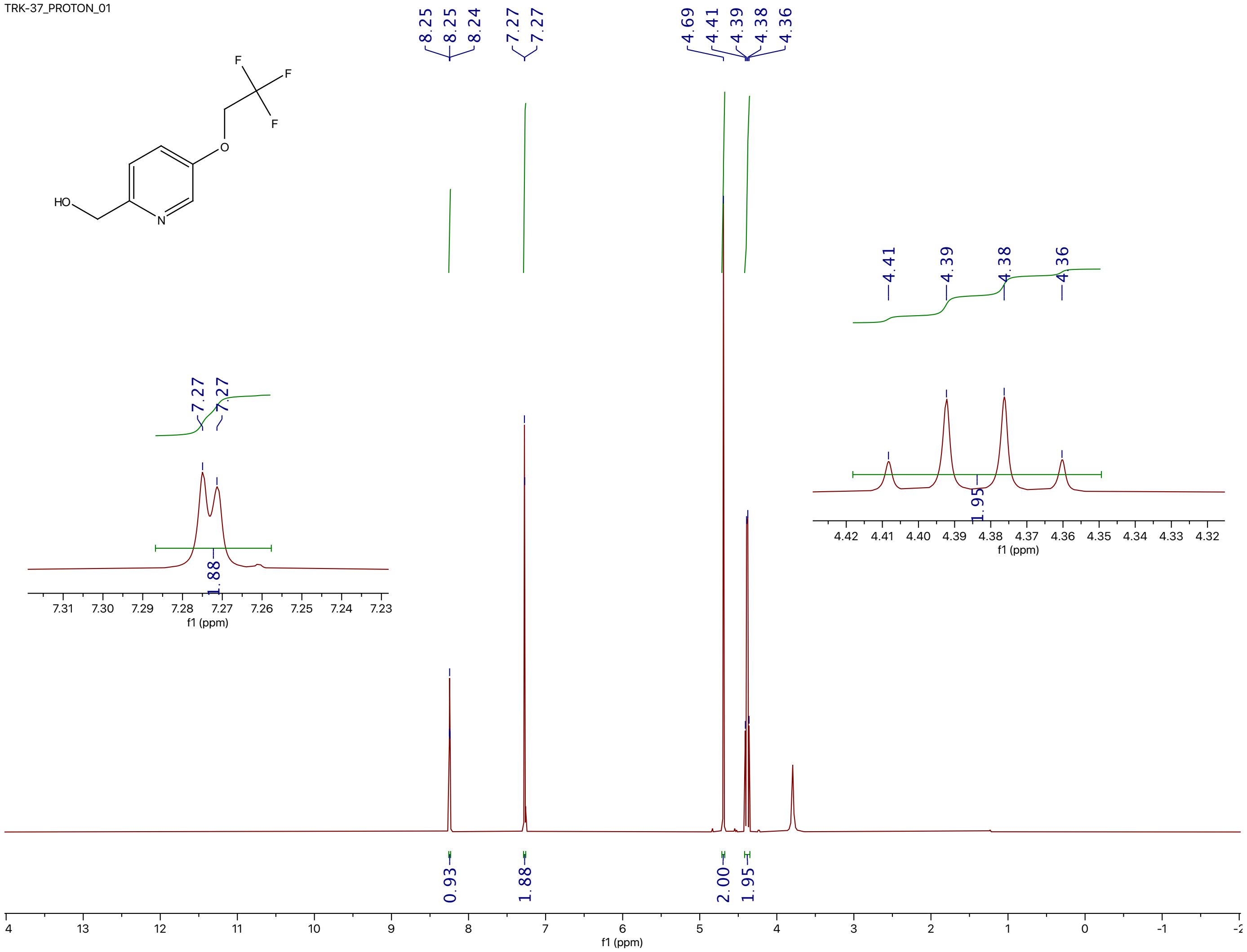

Supplemental Figure 12: ^{1}H NMR of **3**

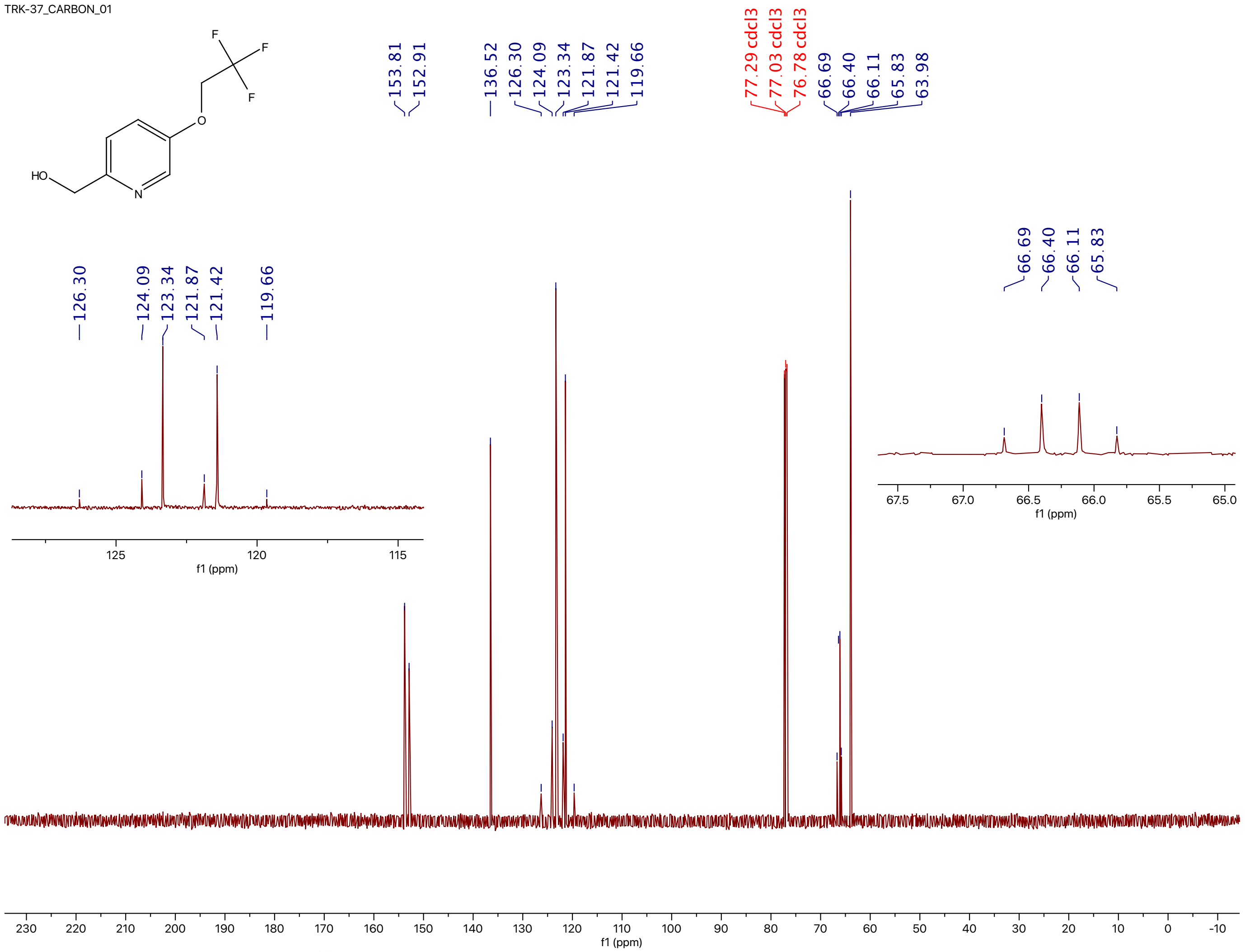

Supplemental Figure 13: ^{13}C NMR of **3**

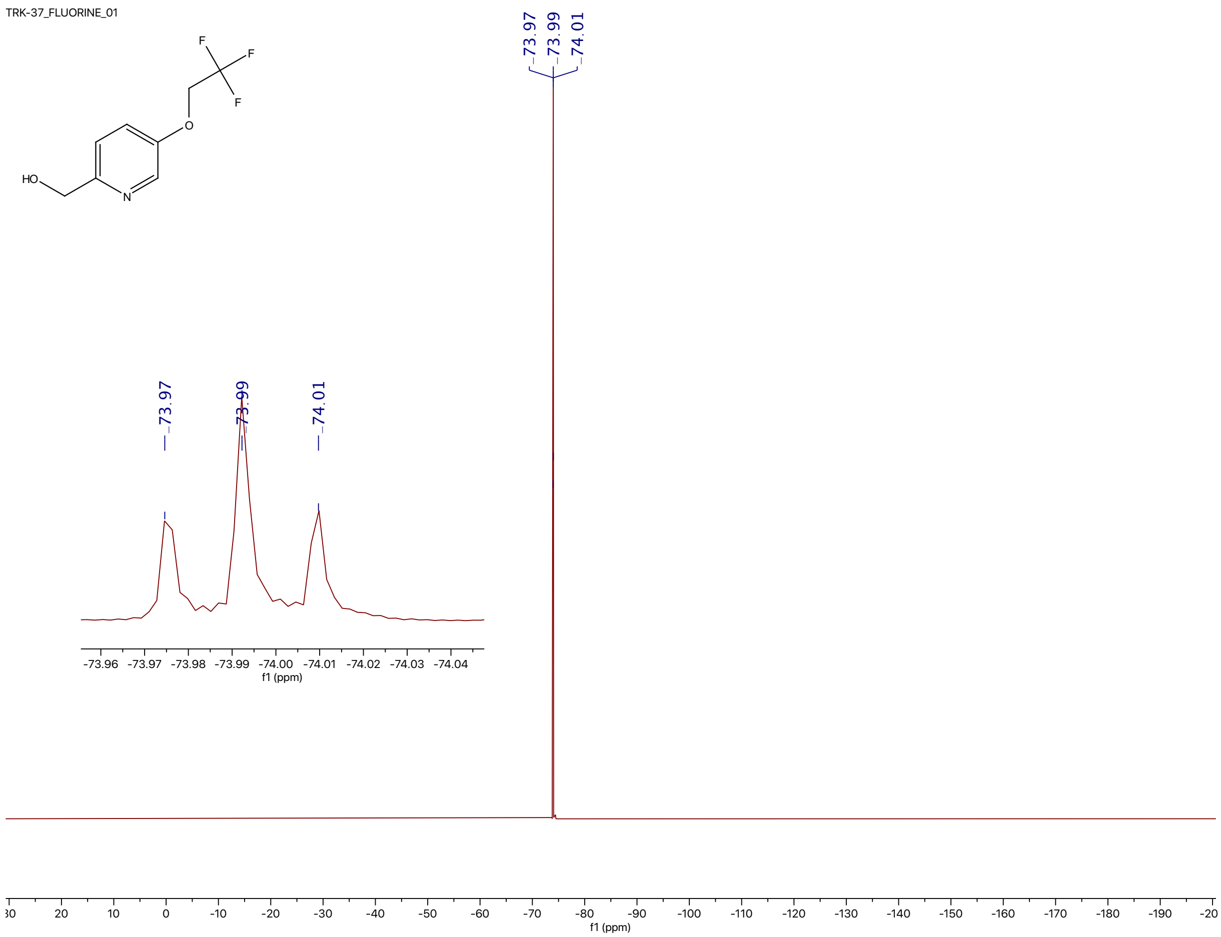

Supplemental Figure 14: ^{19}F NMR of **3**

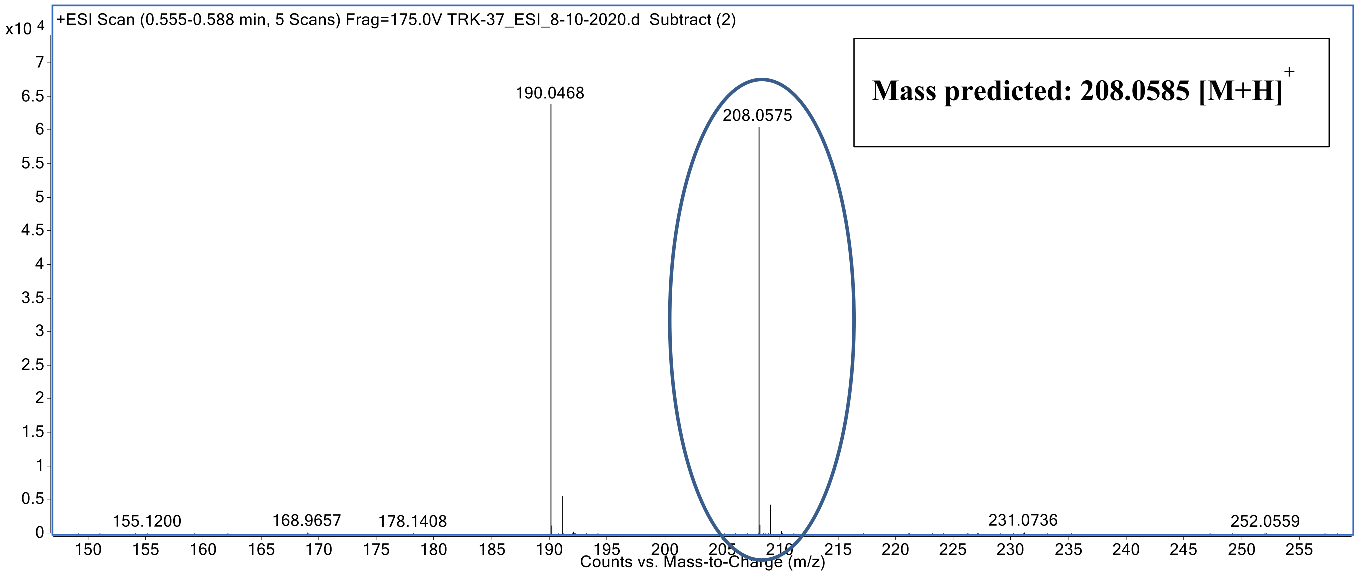

Supplemental Figure 15: HRMS of **3**

3.2.2-Methyl-5-((5-(2,2,2-trifluoroethoxy)pyridin-2-yl)methoxy)benzo[*d*]oxazole (5):

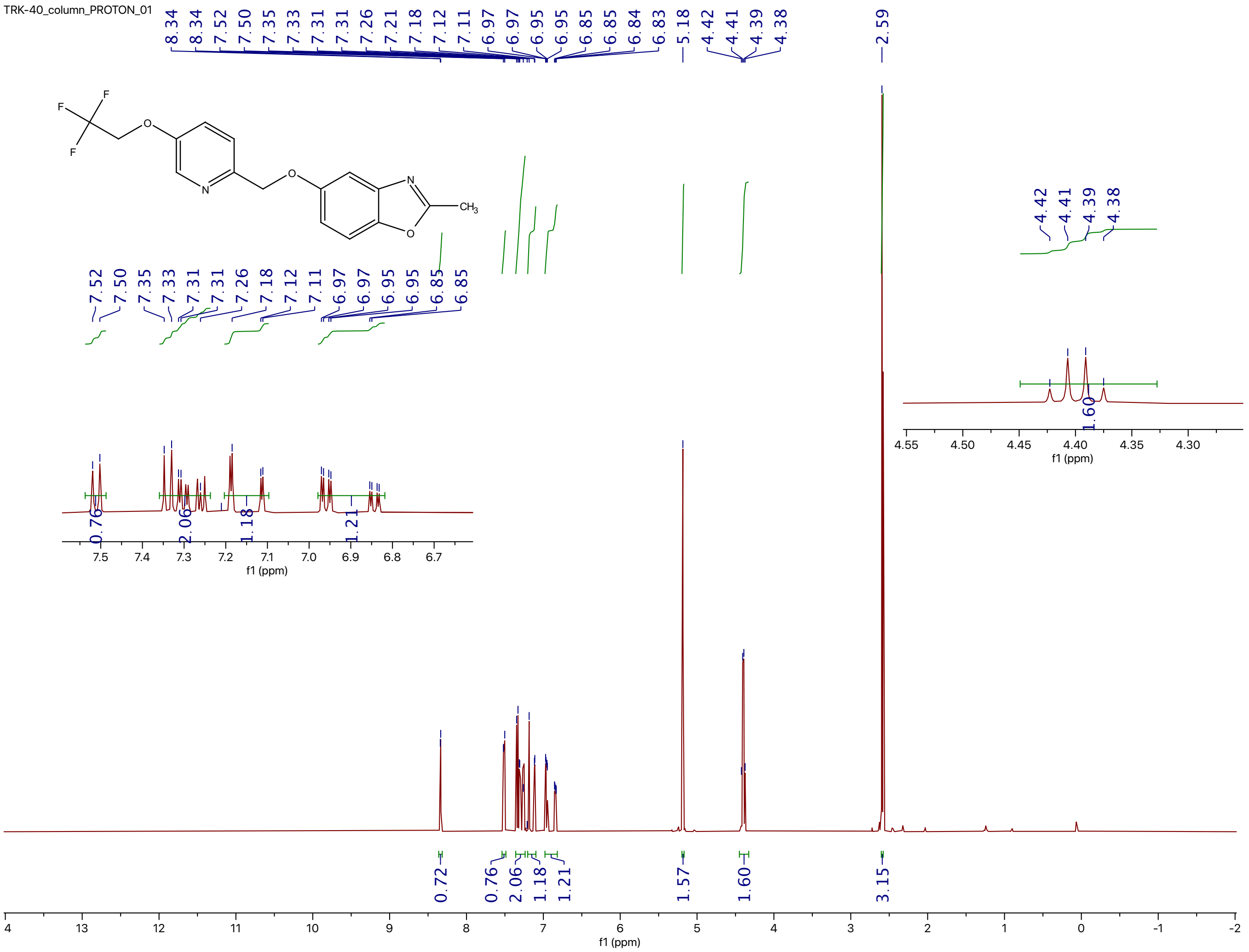

Supplemental Figure 16: ^{1}H NMR of **5**

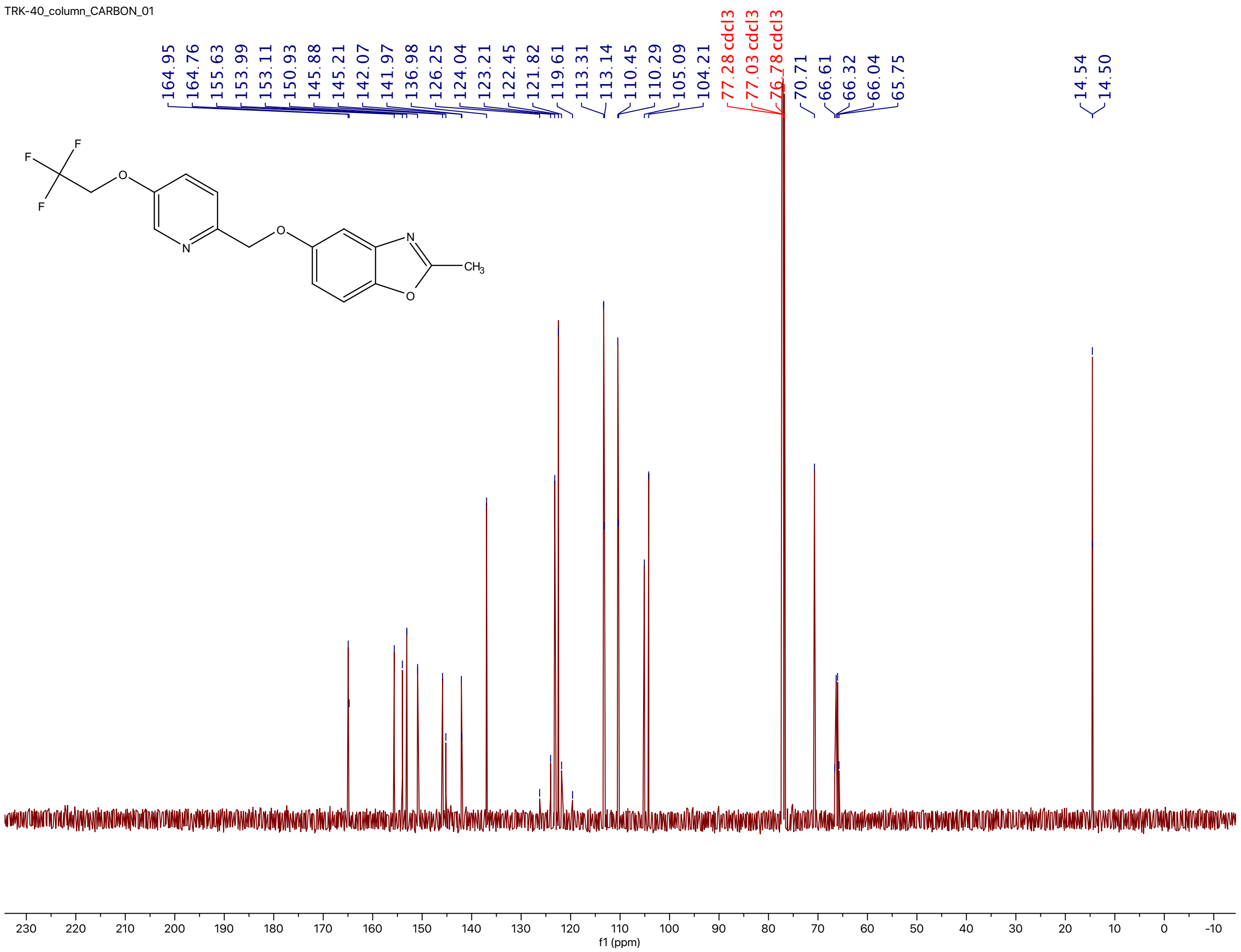

Supplemental Figure 17: ^{13}C NMR of **5**

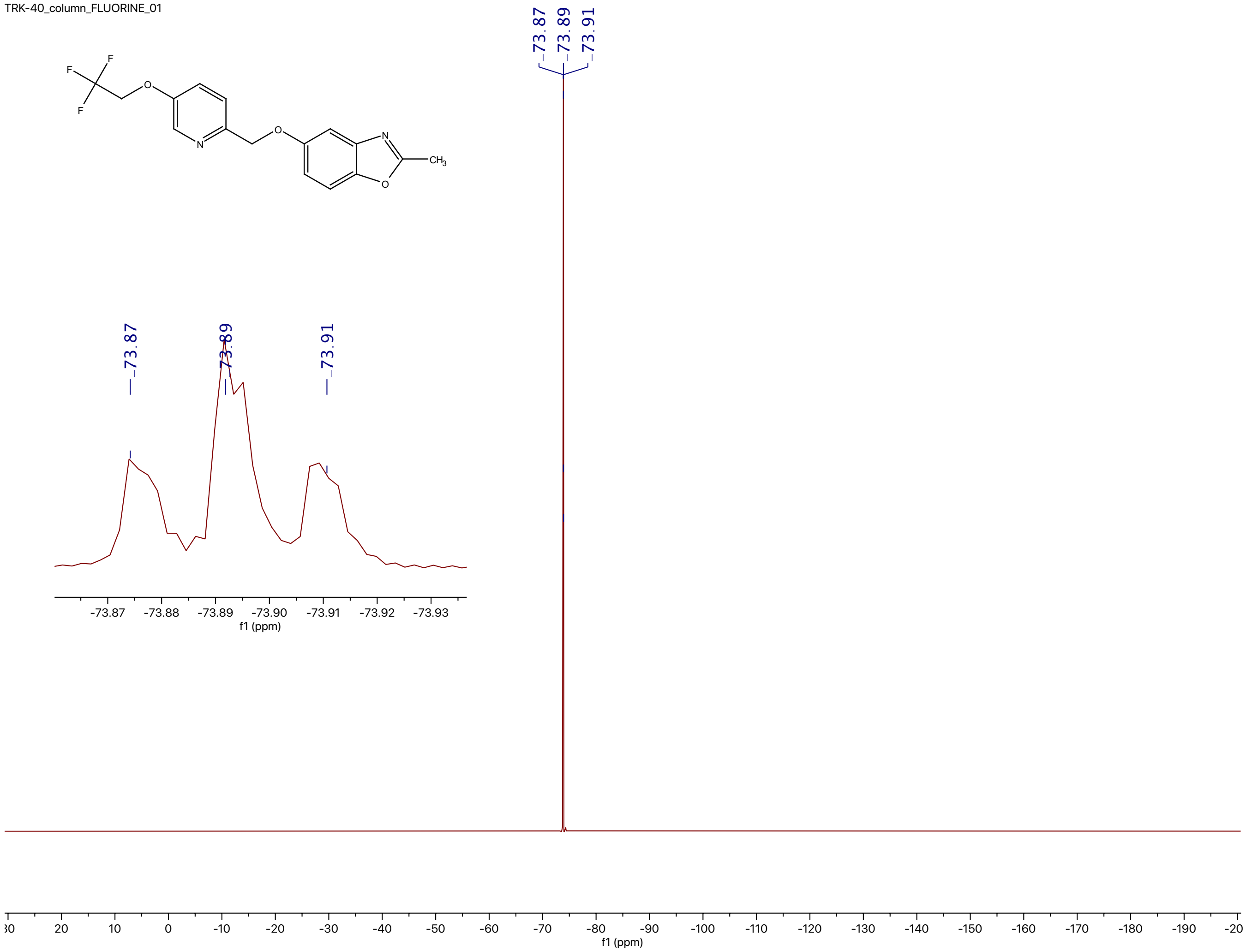

Supplemental Figure 18: ^{19}F NMR of **5**

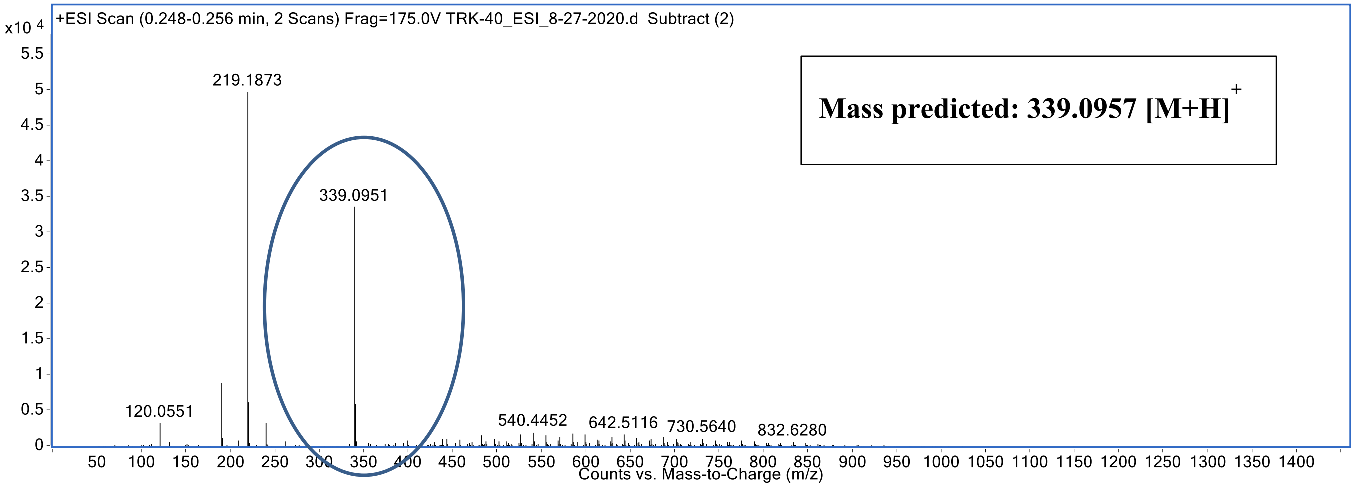

Supplemental Figure 19: HRMS of **5**

3.3.2-Amino-4-((5-(2,2,2-trifluoroethoxy)pyridin-2-yl)methoxy)phenol (7):

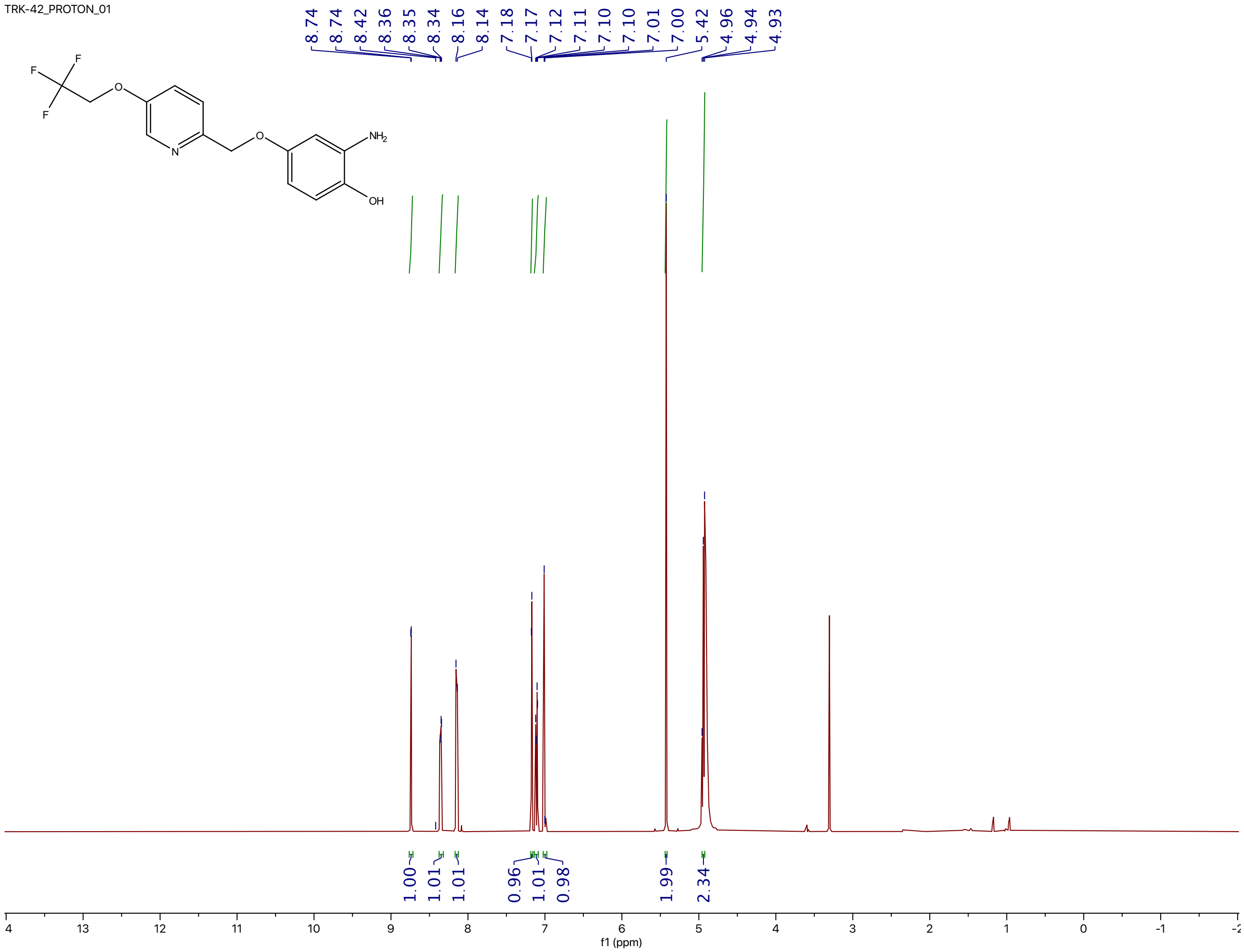

Supplemental Figure 20: ^{1}H NMR of **7**

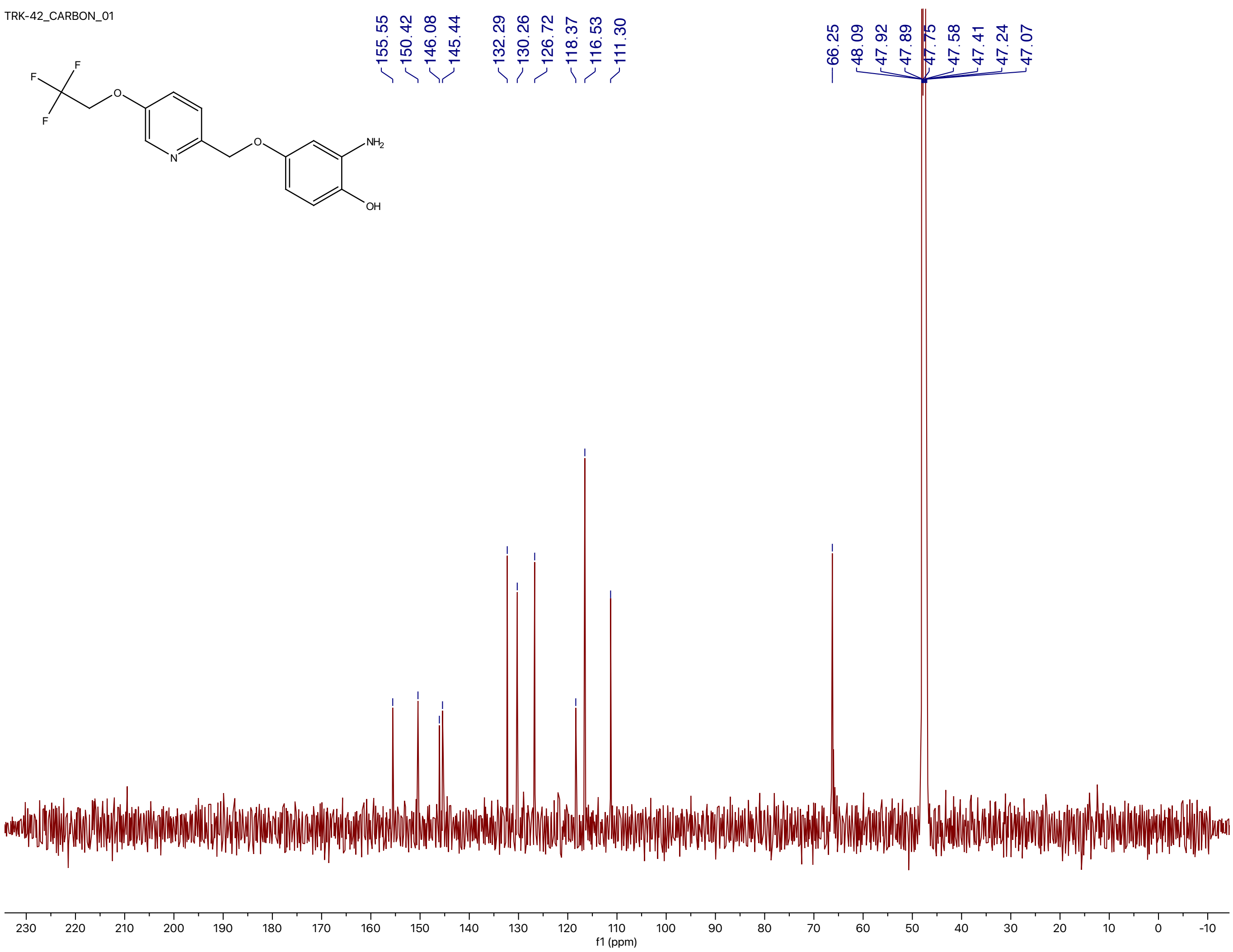

Supplemental Figure 21: ^{13}C NMR of **7**

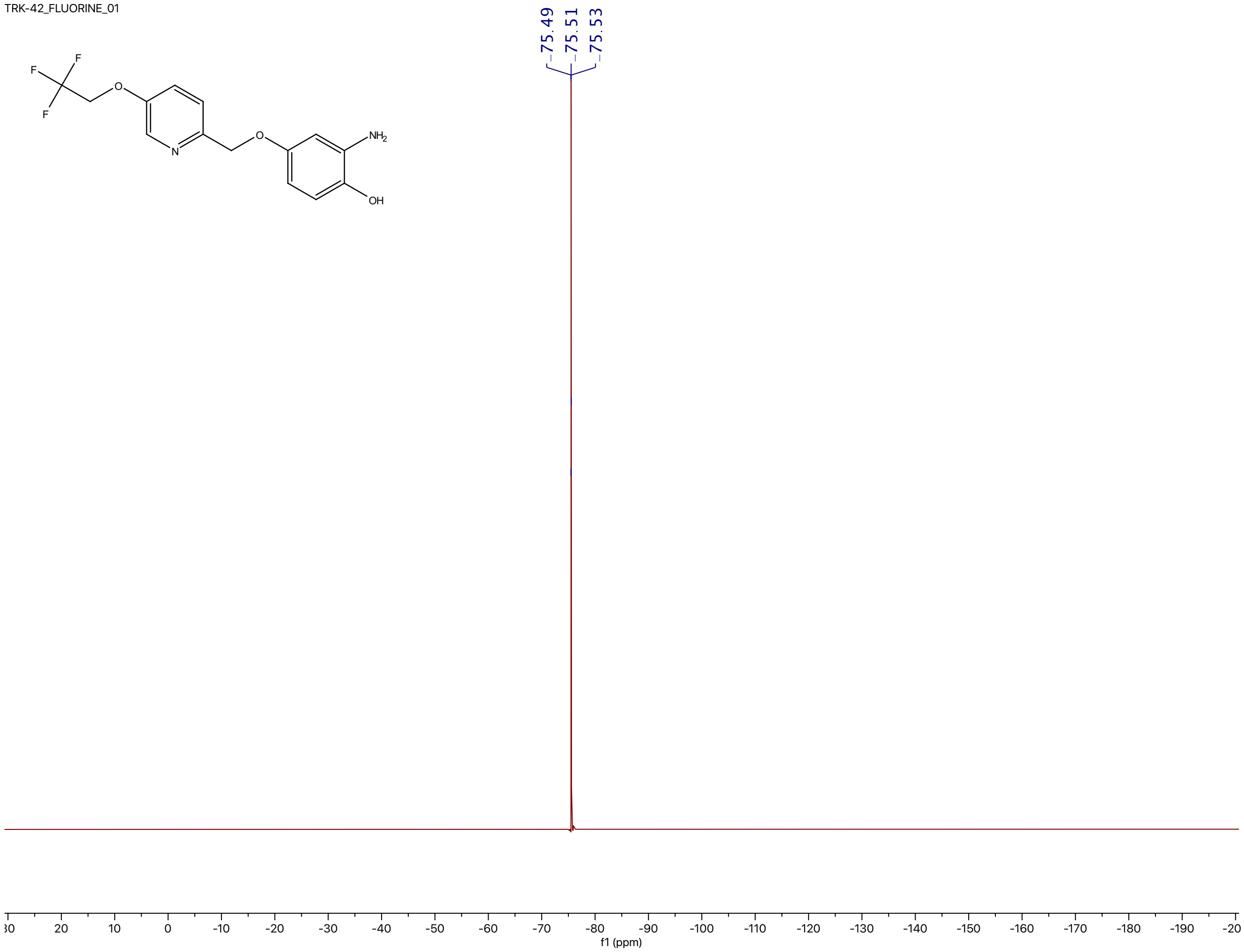

Supplemental Figure 22: ^{19}F NMR of **7**

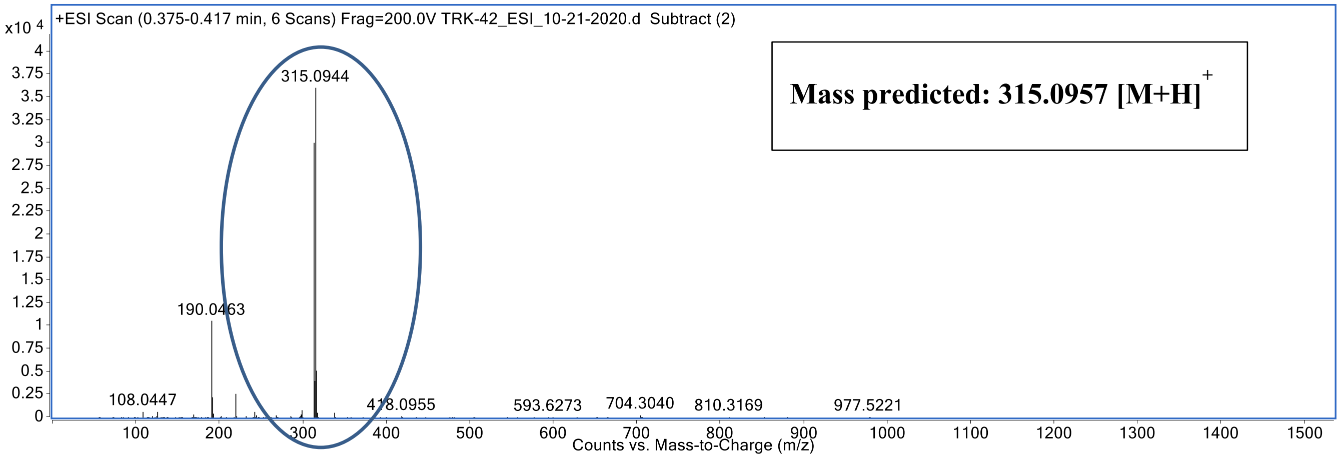

Supplemental Figure 23: HRMS of **7**

3.4. *N*-(2-hydroxy-5-((5-(2,2,2-trifluoroethoxy)pyridin-2-yl)methoxy)phenyl)-1-methyl-6-oxo-1,6-dihydropyridazine-3-carboxamide (9):

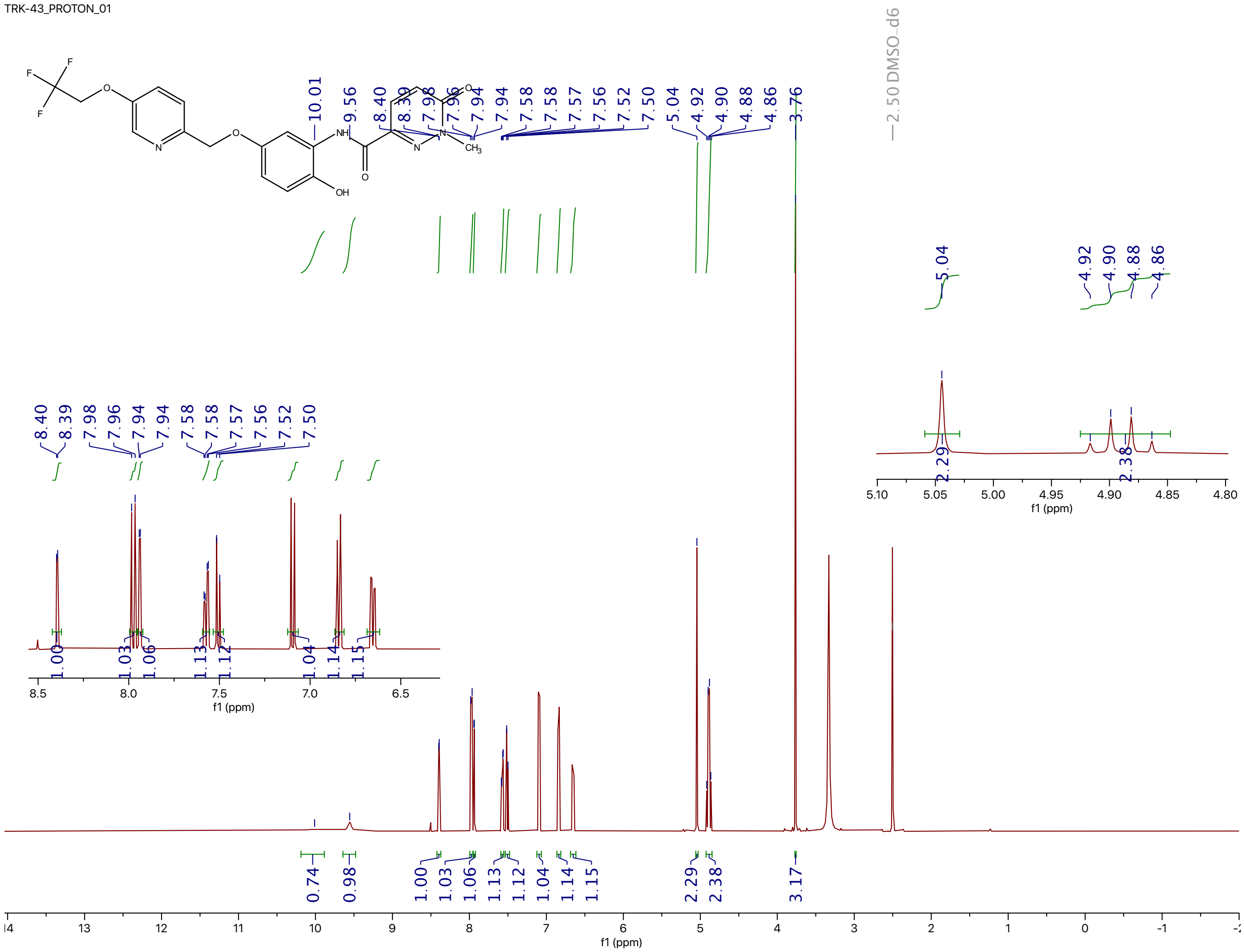

Supplemental Figure 24: ^{1}H NMR of **9**

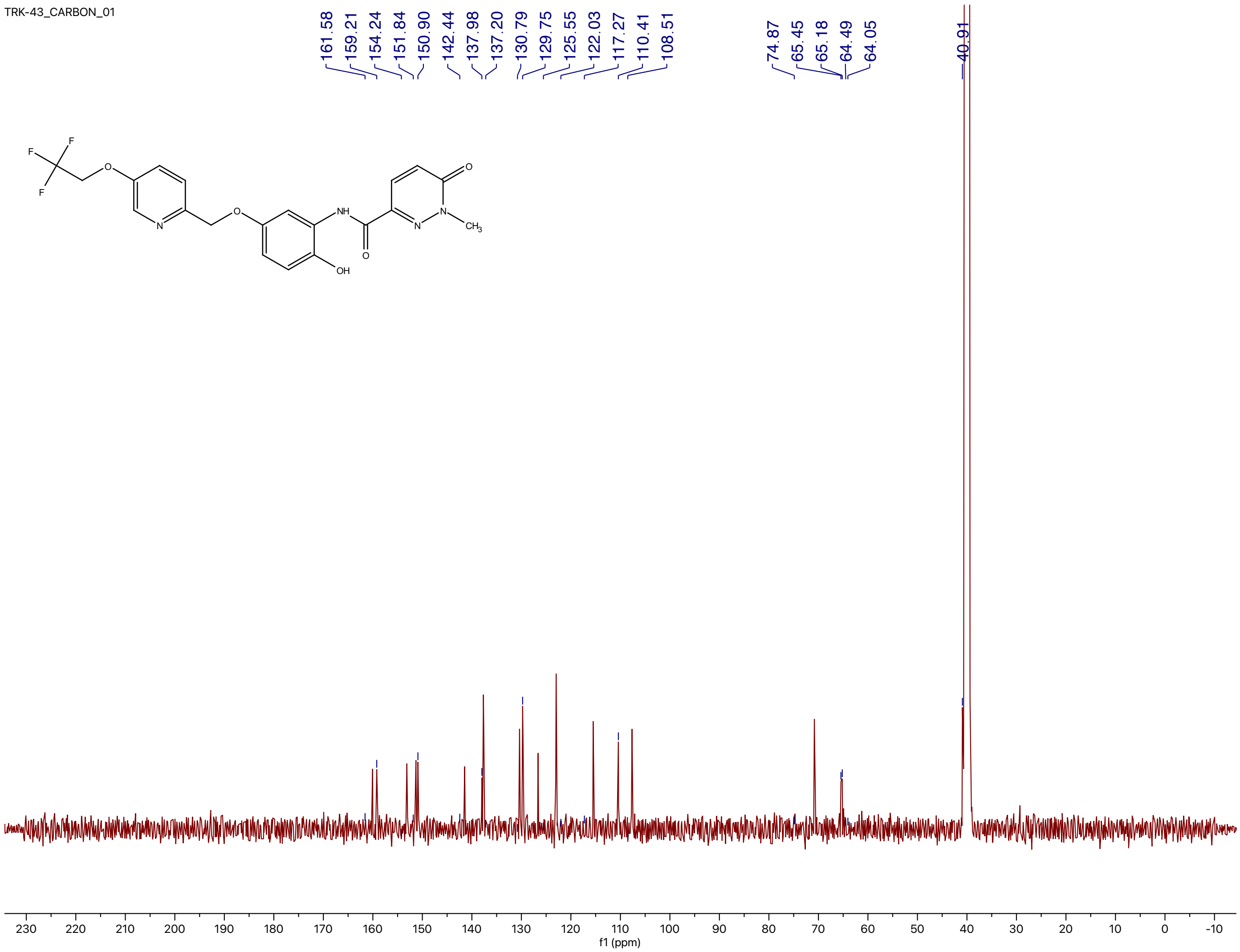

Supplemental Figure 25: ^{13}C NMR of **9**

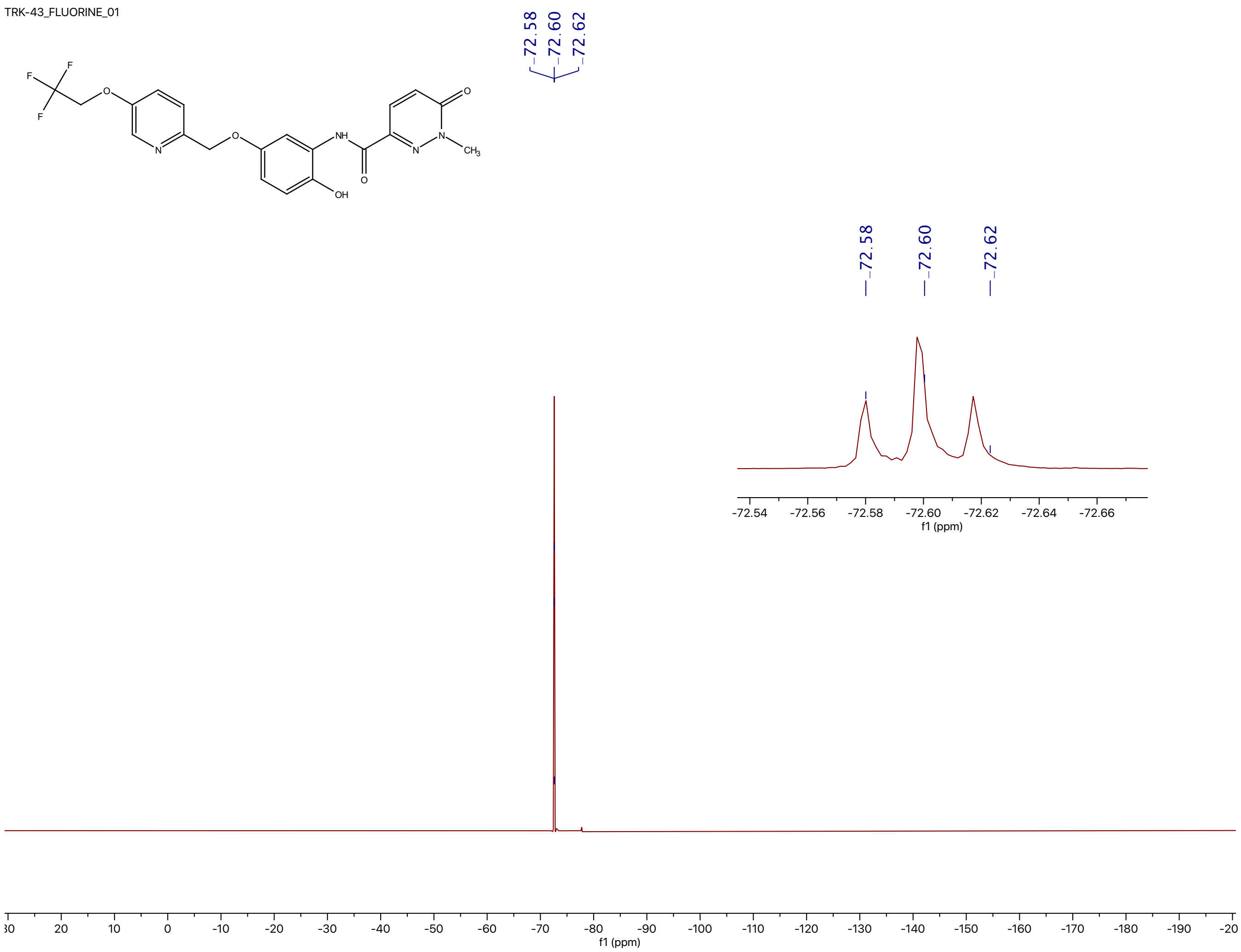

Supplemental Figure 26: ^{19}F NMR of **9**

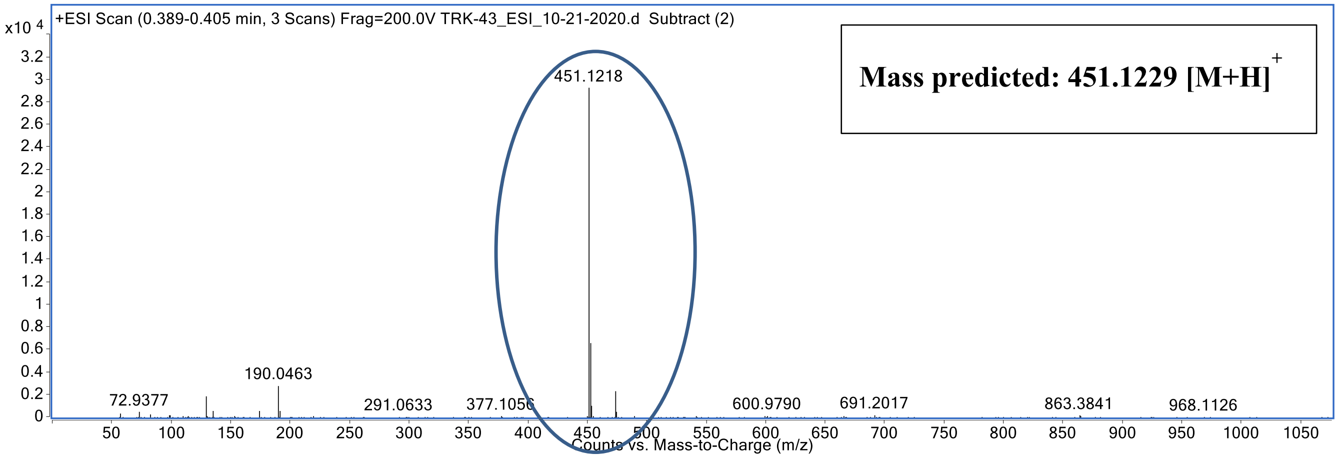

Supplemental Figure 27: HRMS of **9**

3.5.2-Methyl-6-(5-((5-(2,2,2-trifluoroethoxy)pyridin-2-yl)methoxy)benzo[d]oxazol-2-yl)pyridazin-3(2*H*)-one (1):

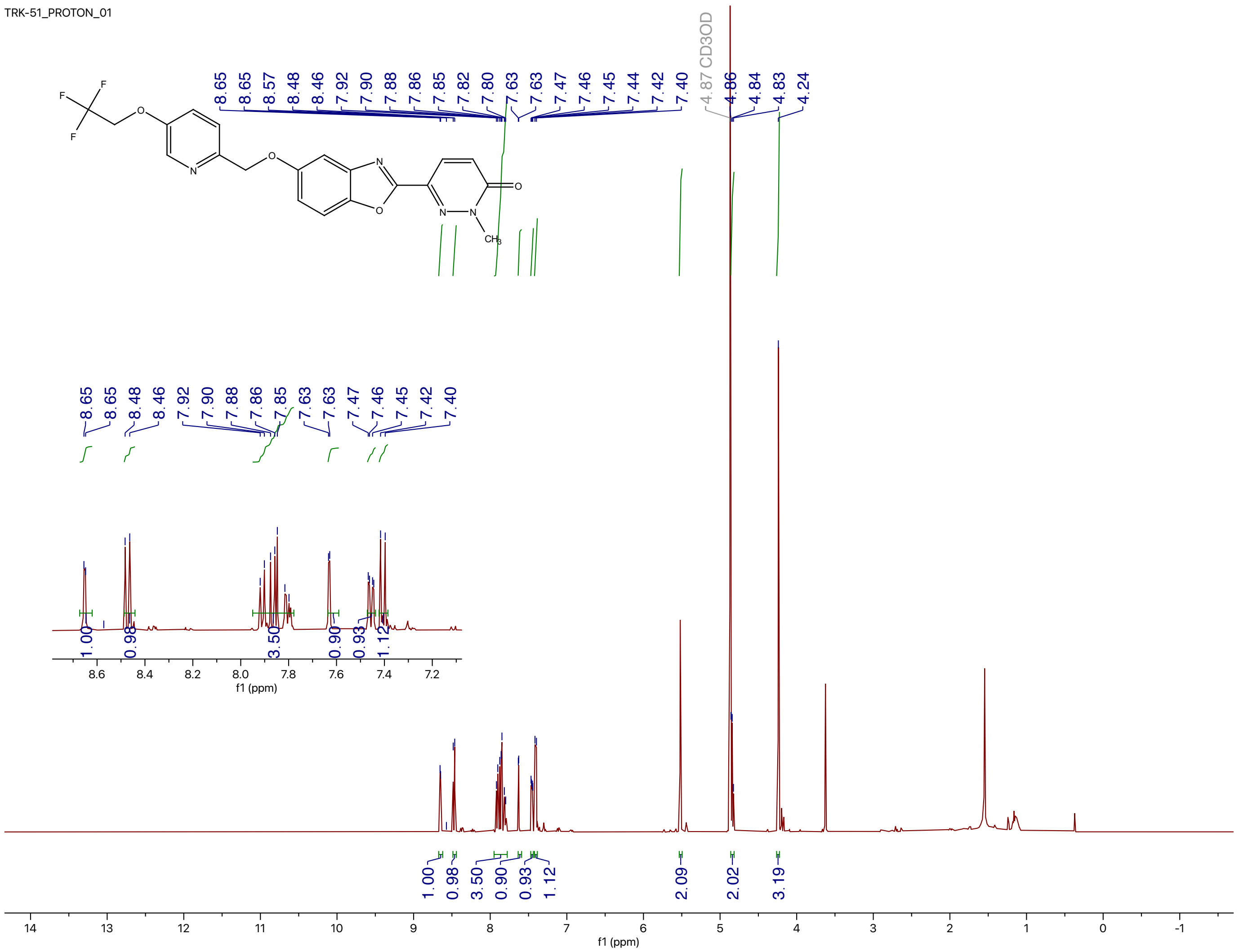

Supplemental Figure 28: ^{1}H NMR of **1**

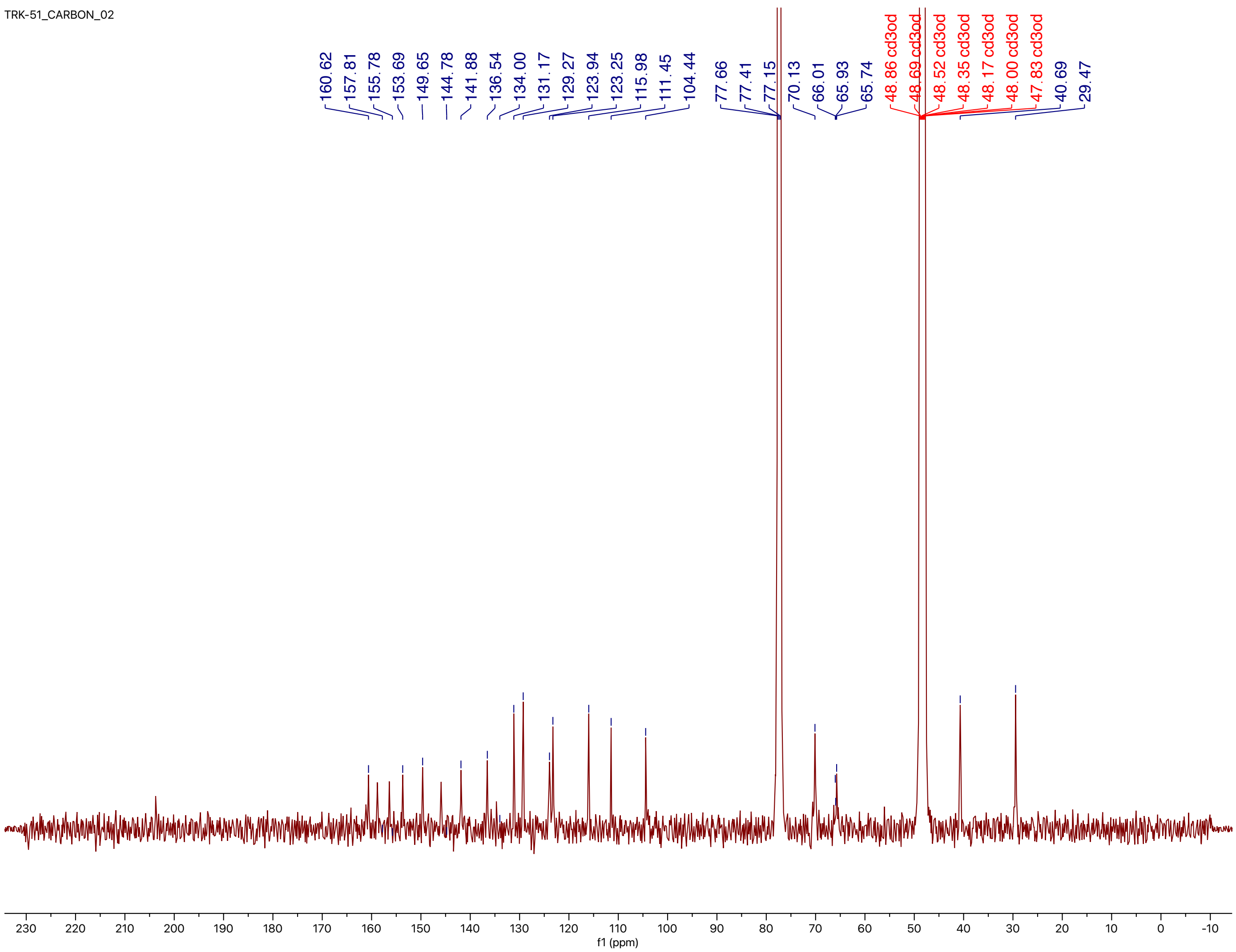

Supplemental Figure 29: ^{13}C NMR of **1**

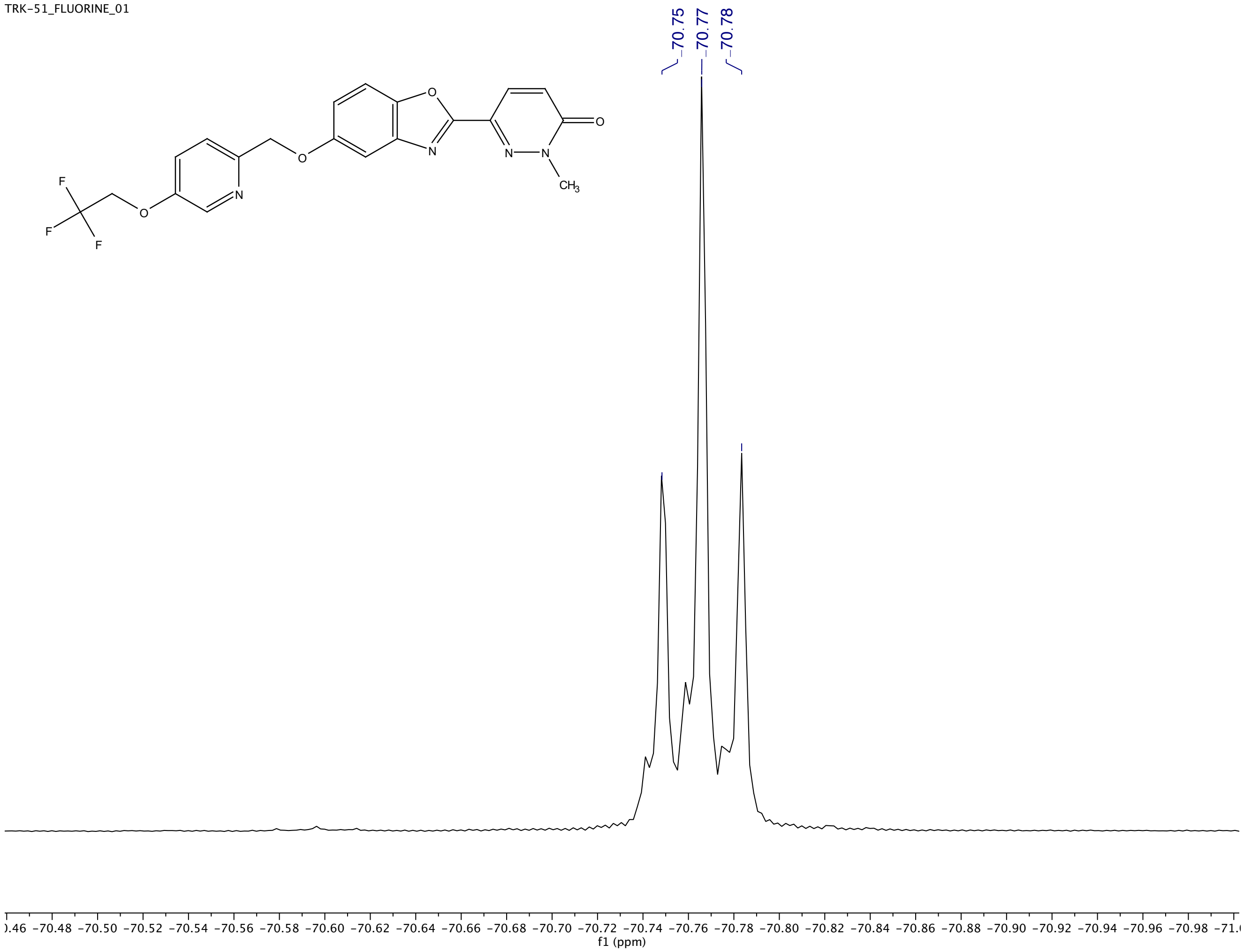

Supplemental Figure 30: ^{19}F NMR of **1**

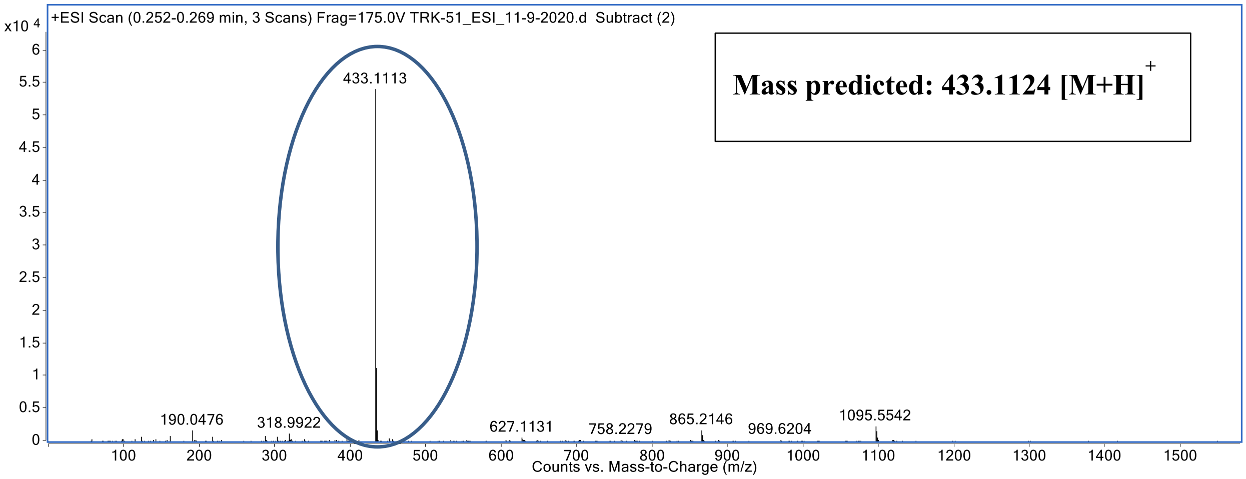

Supplemental Figure 31: HRMS of **1**

3.6. (5-((2,2-Difluorovinyl)oxy)pyridin-2-yl)methanol (4):

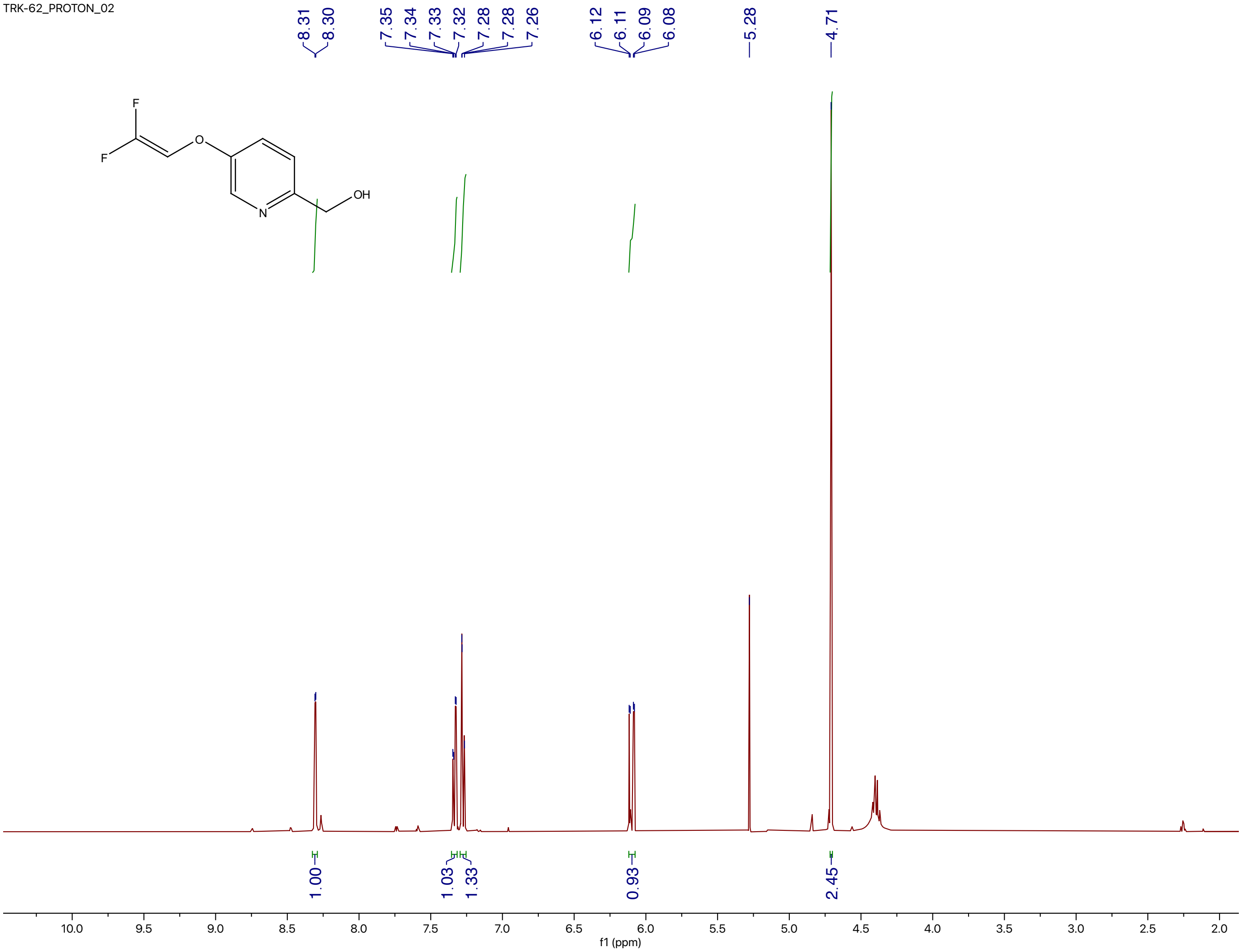

Supplemental Figure 32: ^{1}H NMR of **4**

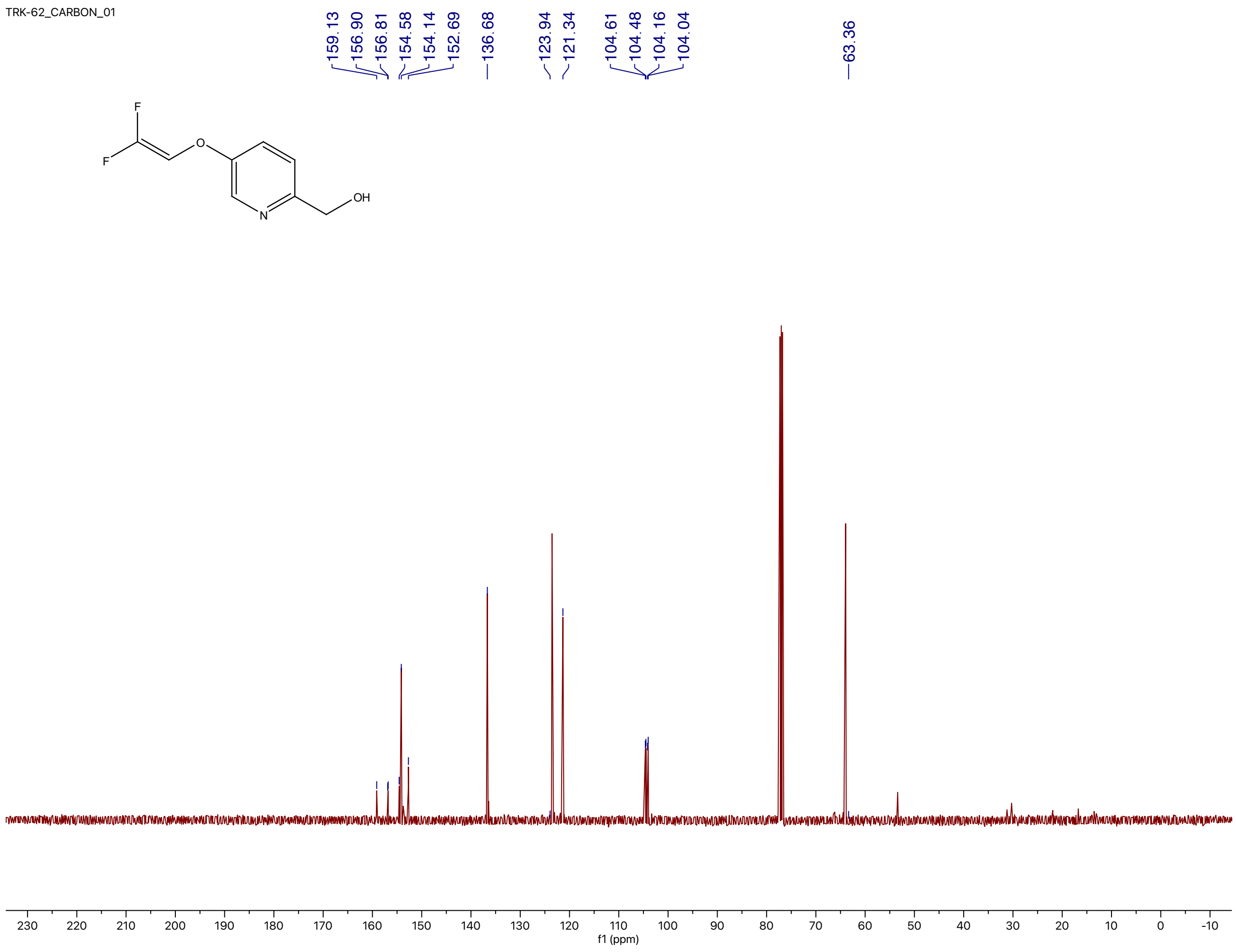

Supplemental Figure 33: ^{13}C NMR of **4**

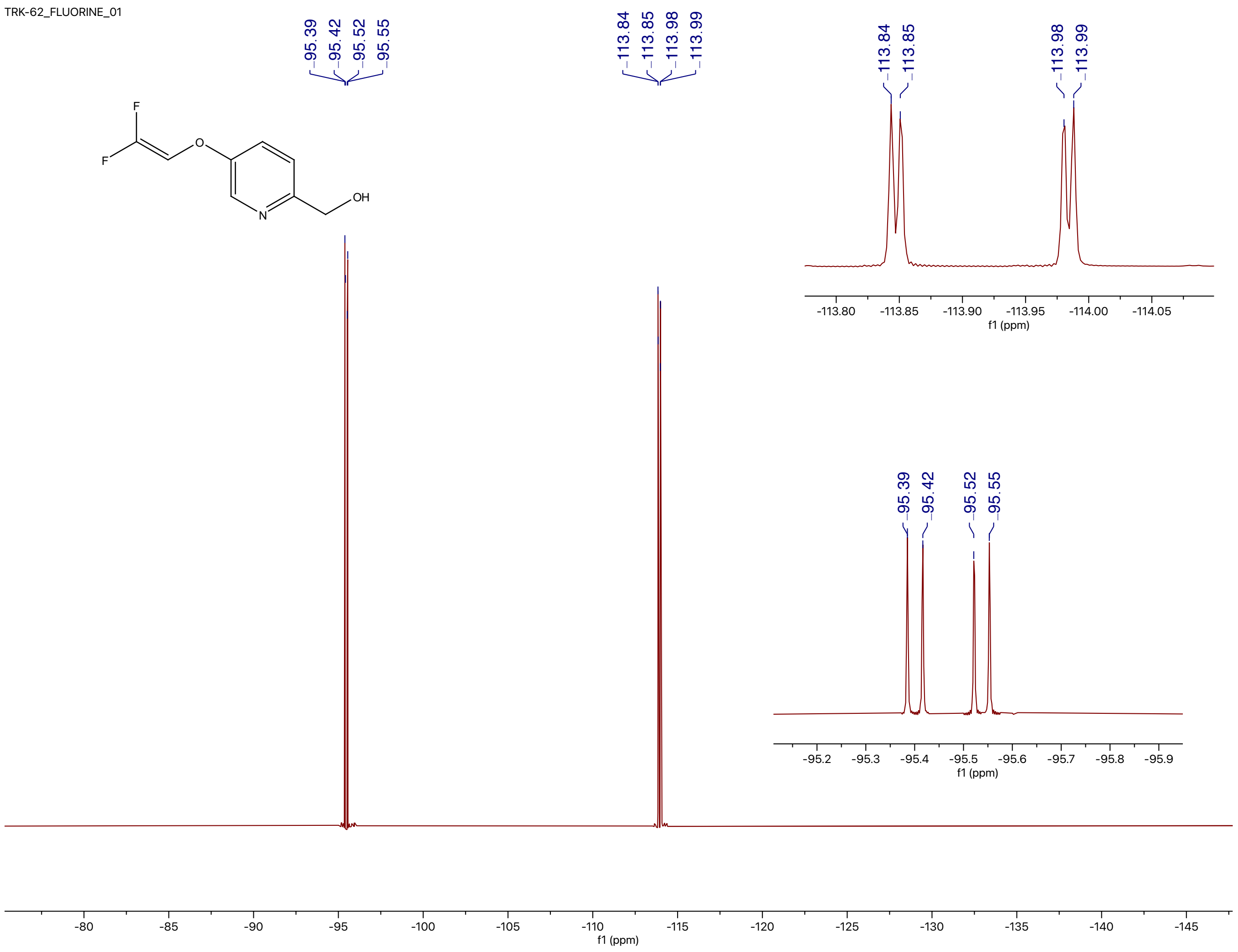

Supplemental Figure 34: ^{19}F NMR of **4**

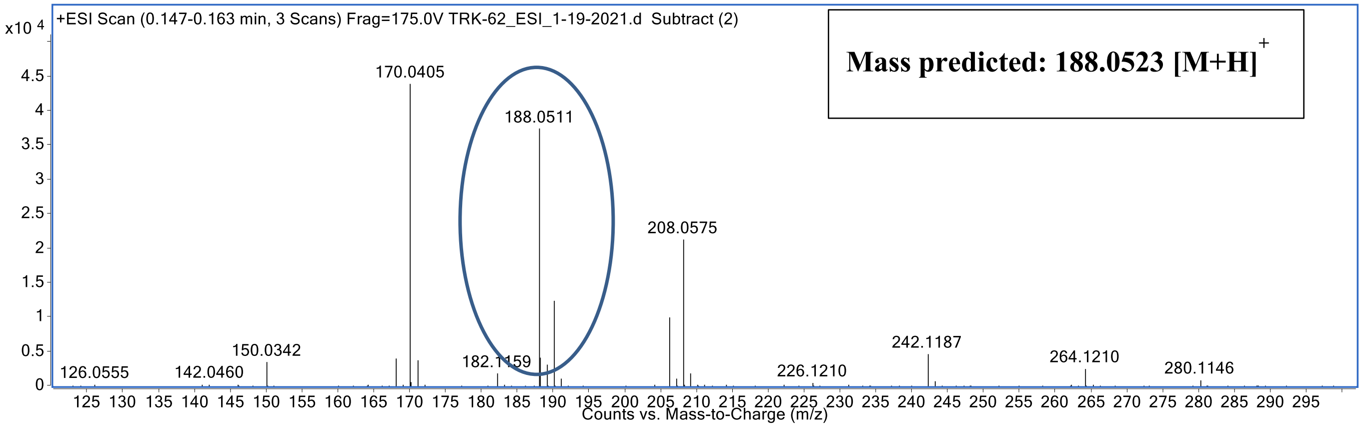

Supplemental Figure 35: HRMS of **4**

3.7. 5-((5-((2,2-Difluorovinyl)oxy)pyridin-2-yl)methoxy)-2-methylbenzo[*d*]oxazole (6):

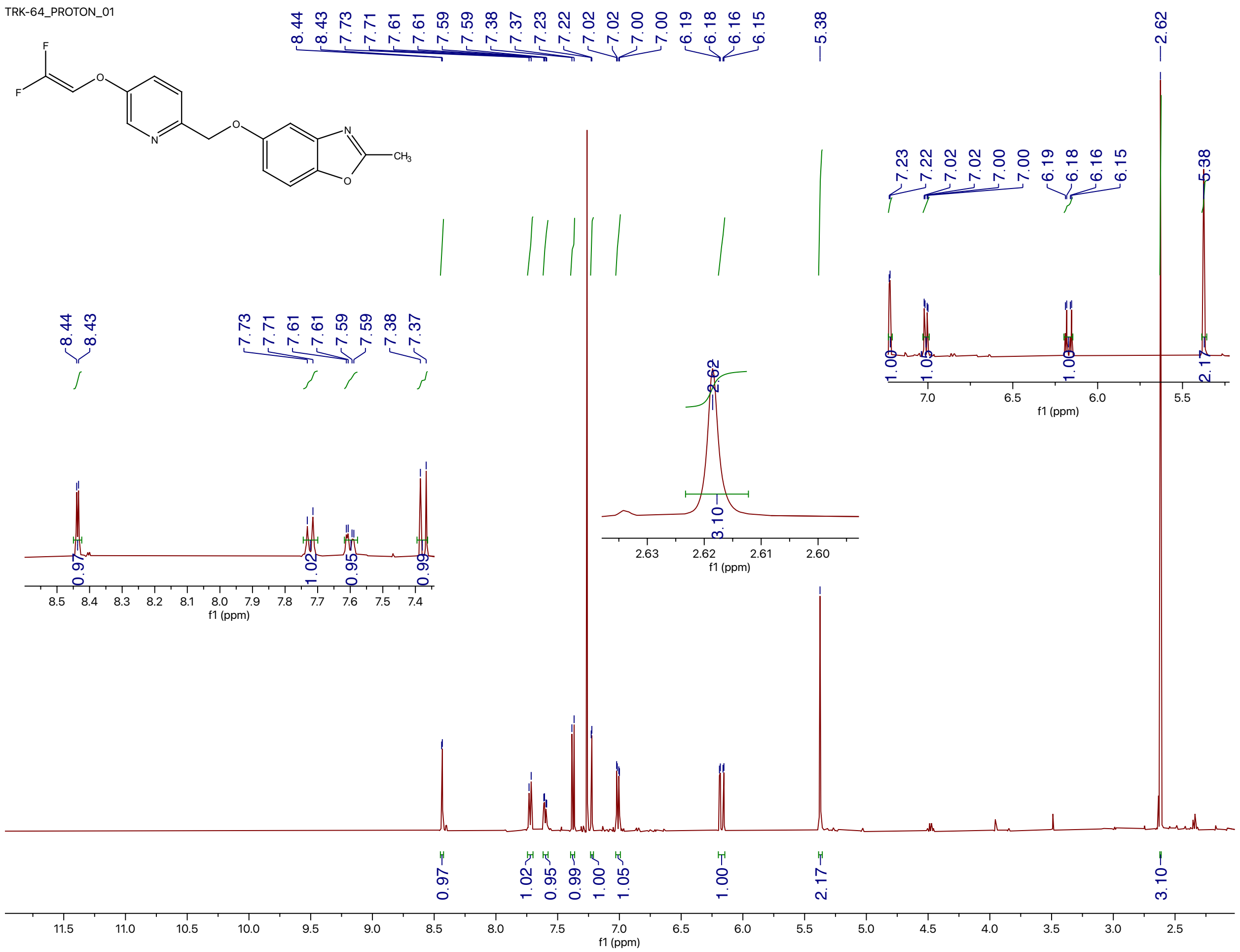

Supplemental Figure 36: ^{1}H NMR of **6**

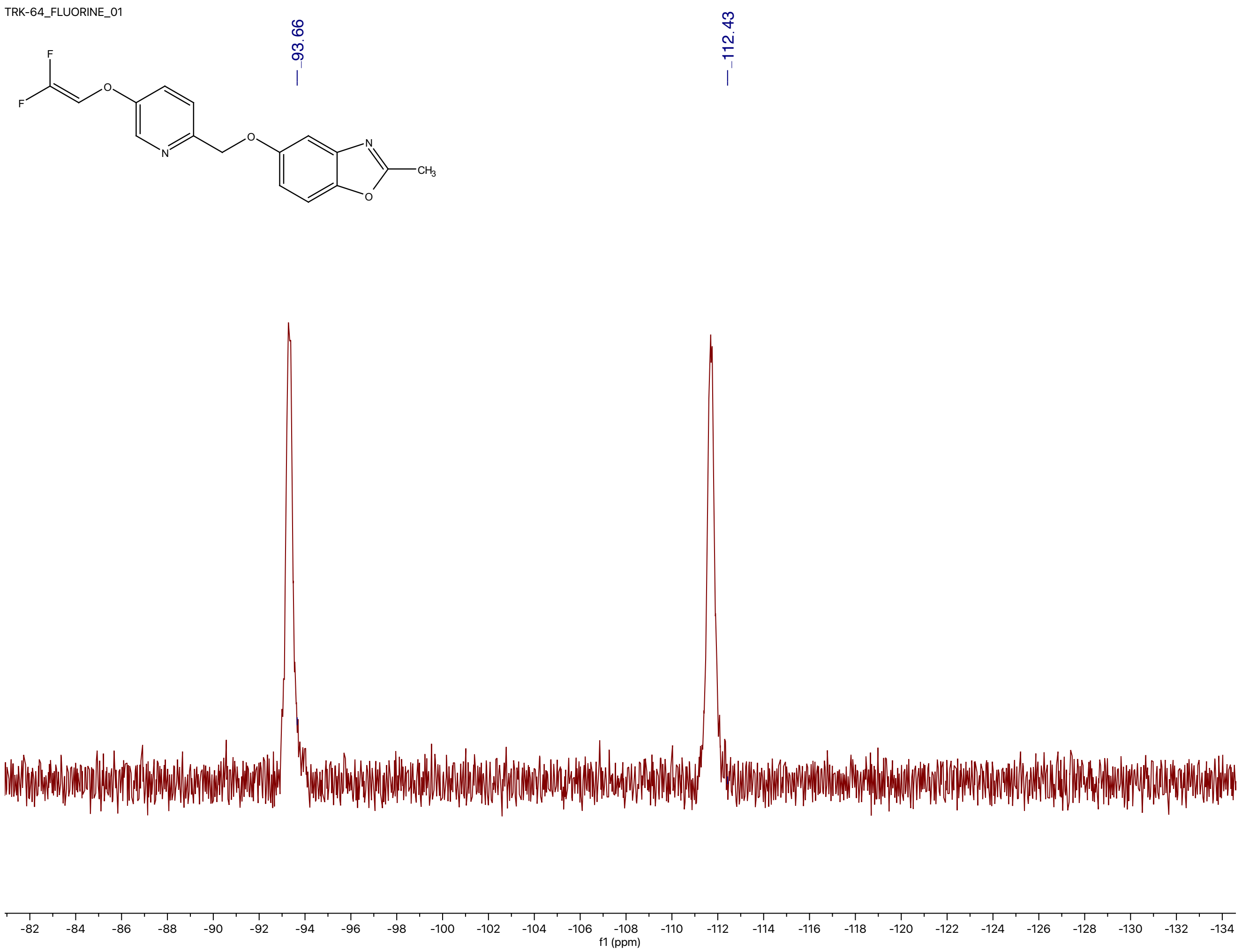

Supplemental Figure 37: ^{19}F NMR of **6**

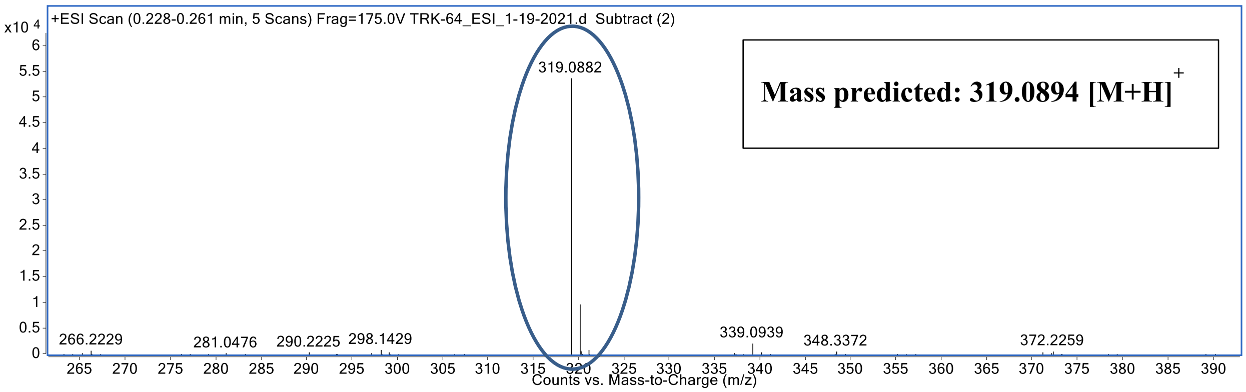

Supplemental Figure 38: HRMS of **6**

3.8. 2-Amino-4-((5-((2,2-difluorovinyl)oxy)pyridin-2-yl)methoxy)phenol (8):

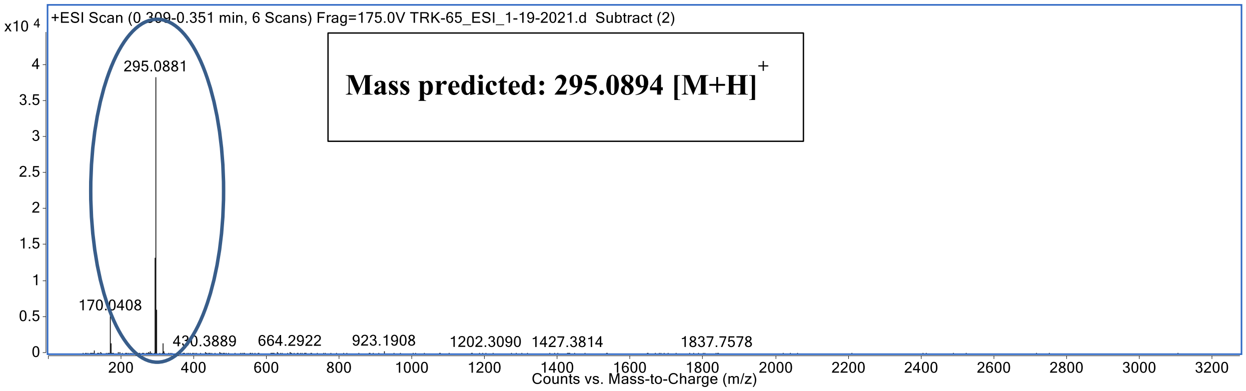

Supplemental Figure 39: HRMS of **8**

3.9. *N*-(5-((5-((2,2-difluorovinyl)oxy)pyridin-2-yl)methoxy)-2-hydroxyphenyl)-1-methyl-6-oxo-1,6-dihydropyridazine-3-carboxamide (10):

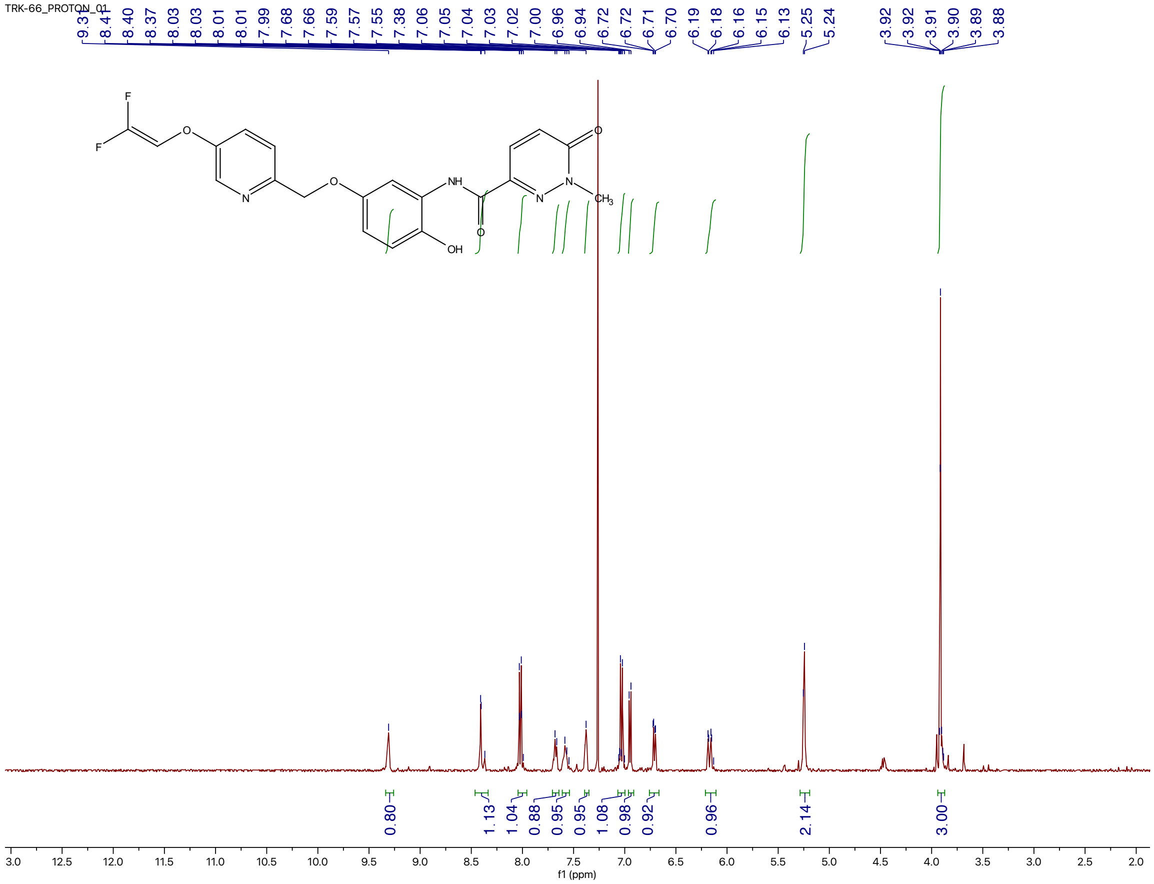

Supplemental Figure 40: ^{1}H NMR of **10**

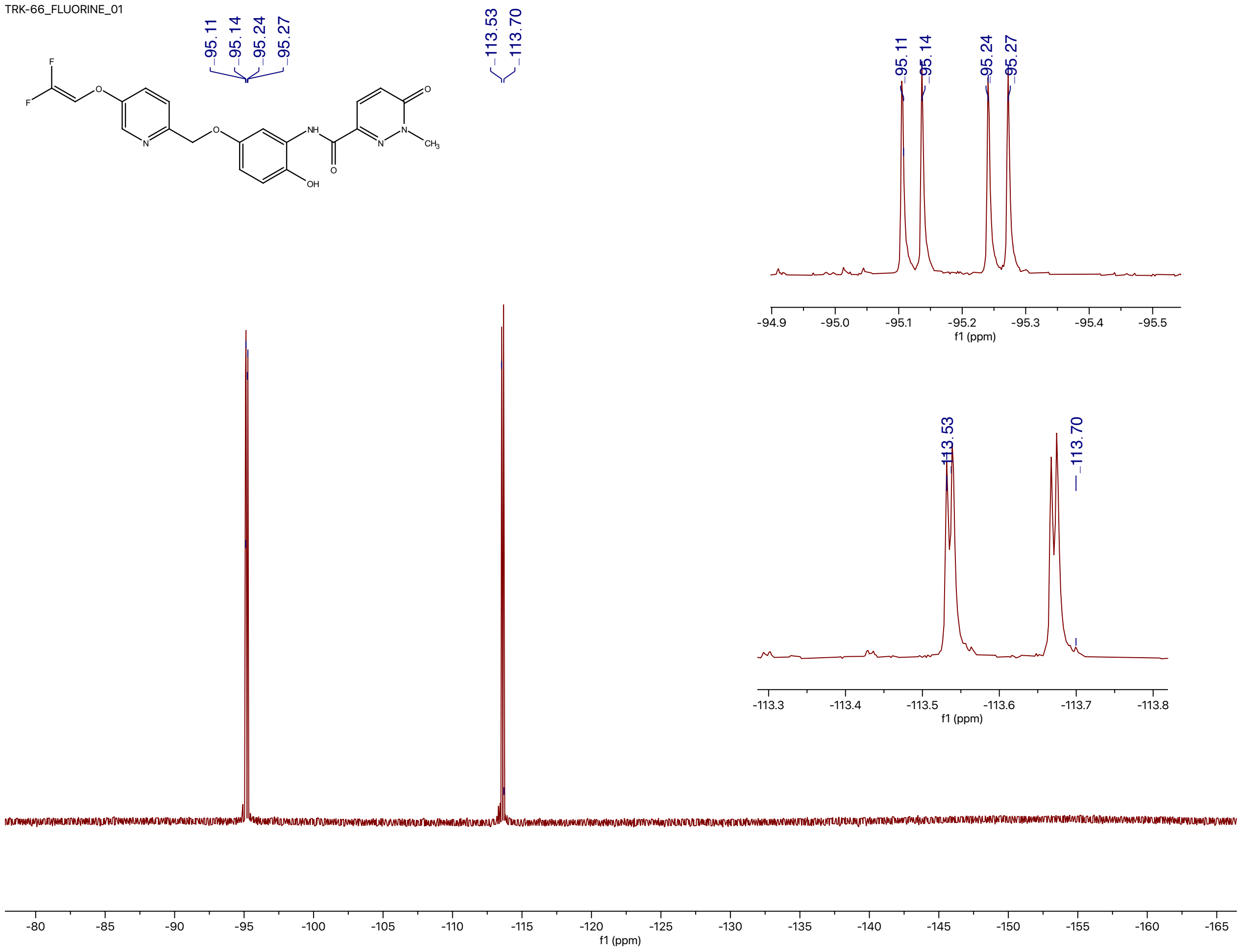

Supplemental Figure 41: ^{19}F NMR of **10**

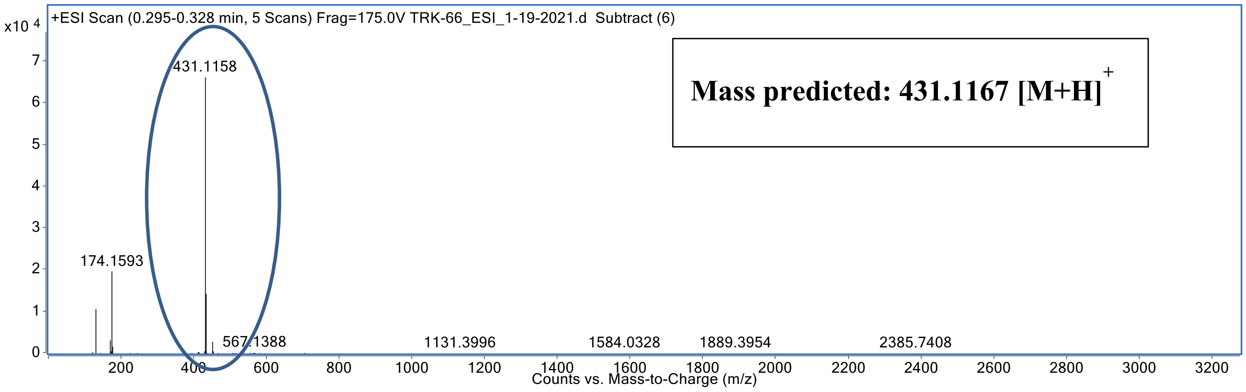

Supplemental Figure 42: HRMS of **10**

3.10. 6-(5-((5-((2,2-Difluorovinyl)oxy)pyridin-2-yl)methoxy)benzo[*d*]oxazol-2-yl)-2-methyl pyridazin-3(2*H*)-one (11):

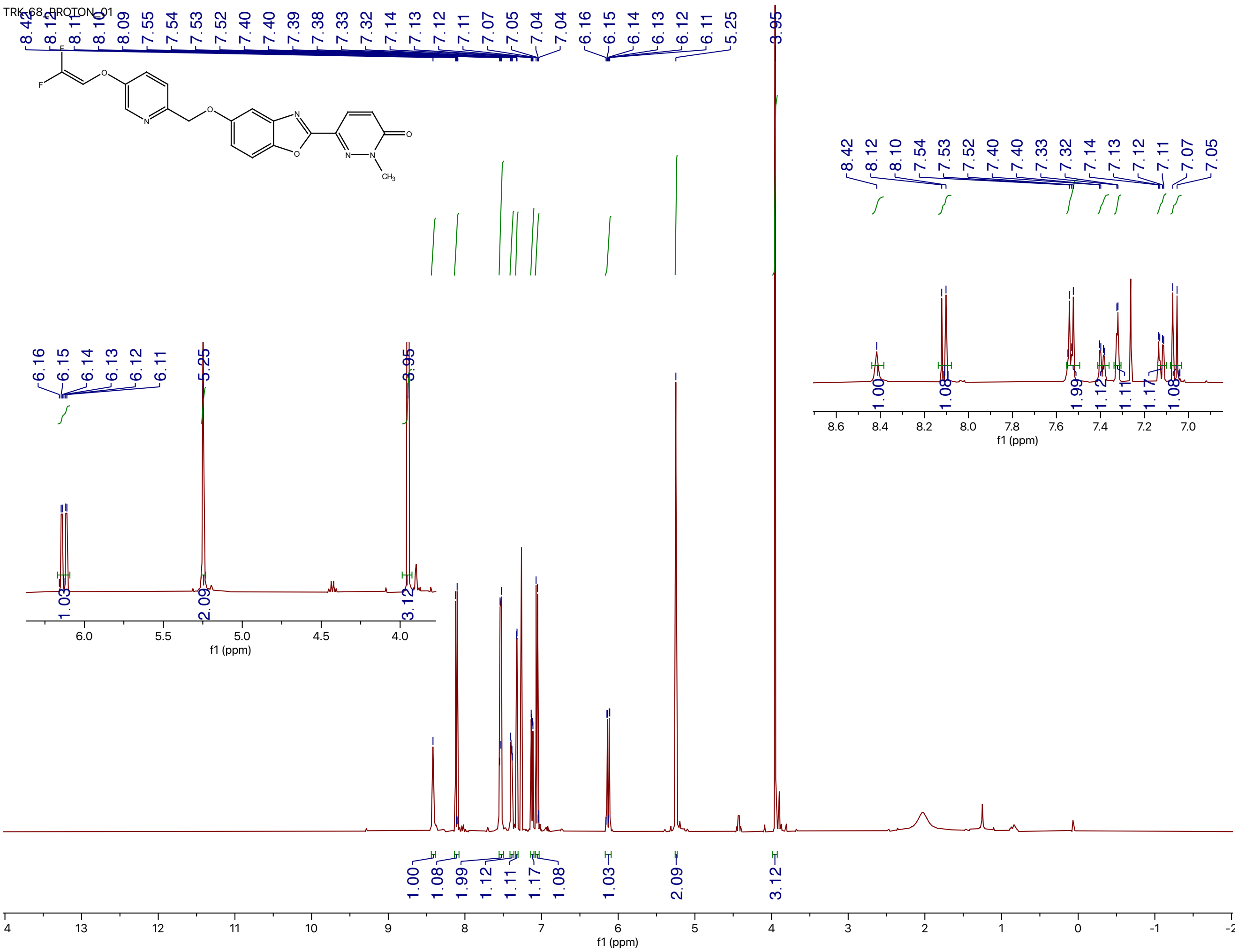

Supplemental Figure 43: ^{1}H NMR of **11**

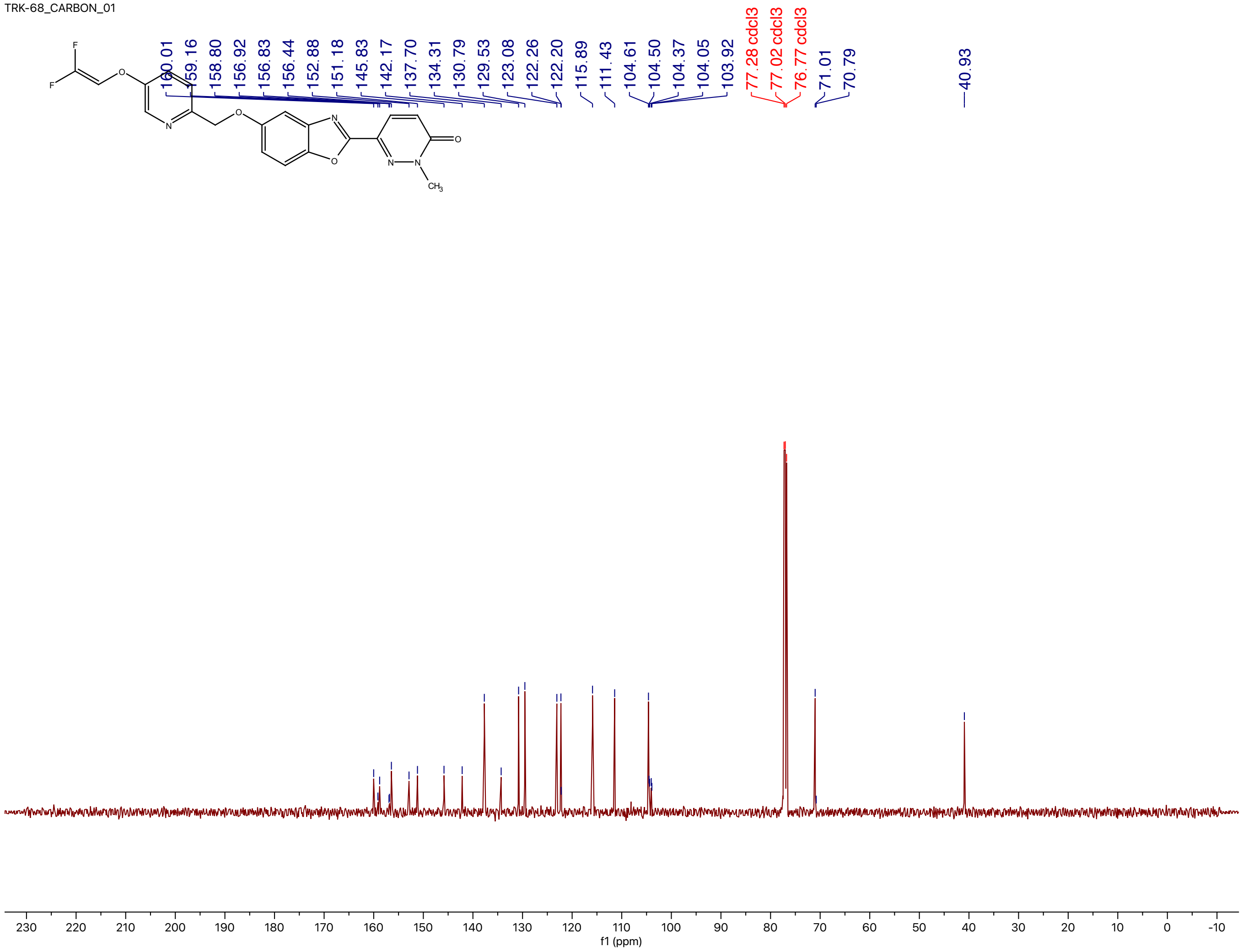

Supplemental Figure 44: ^{13}C NMR of **11**

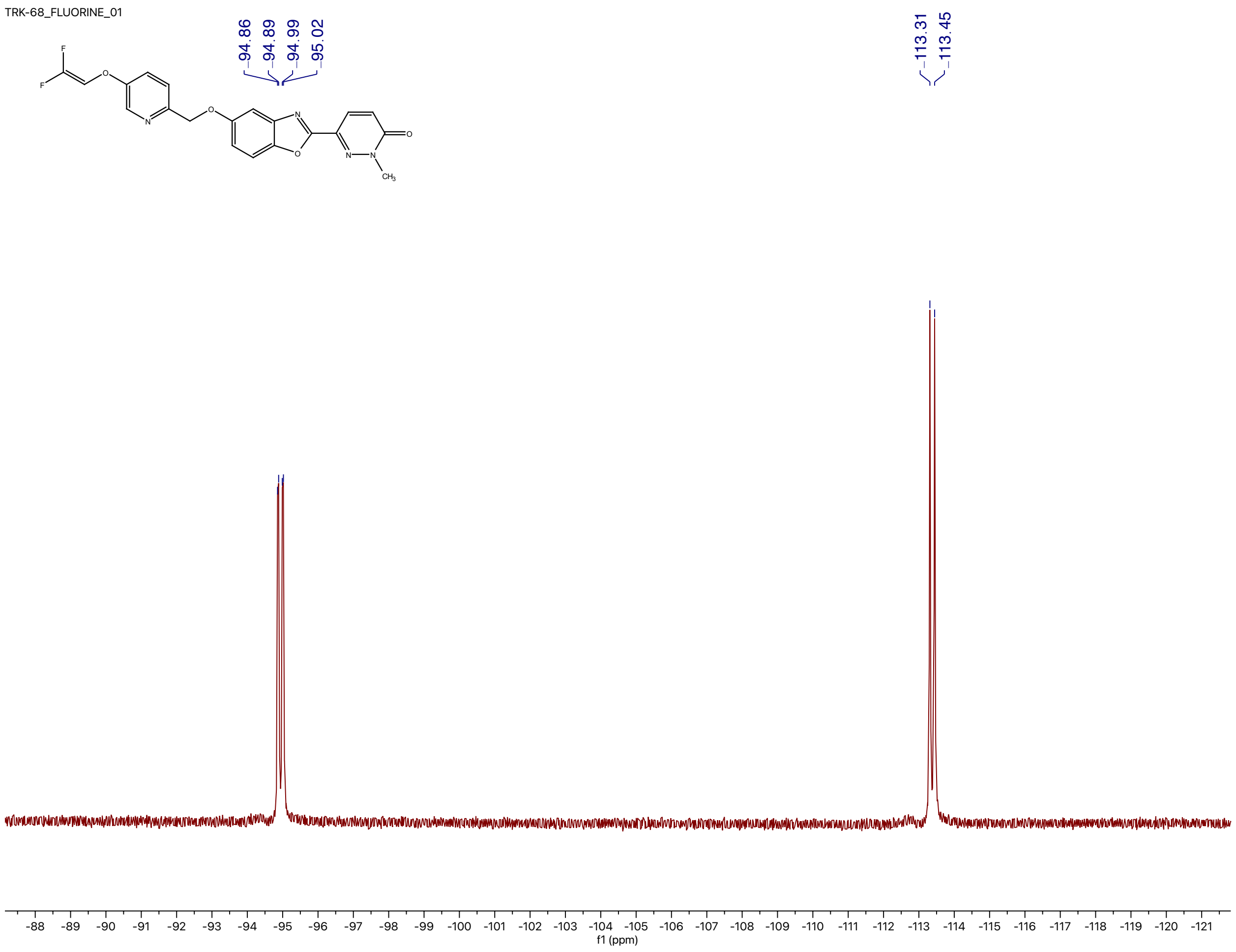

Supplemental Figure 45: ^{19}F NMR of **11**

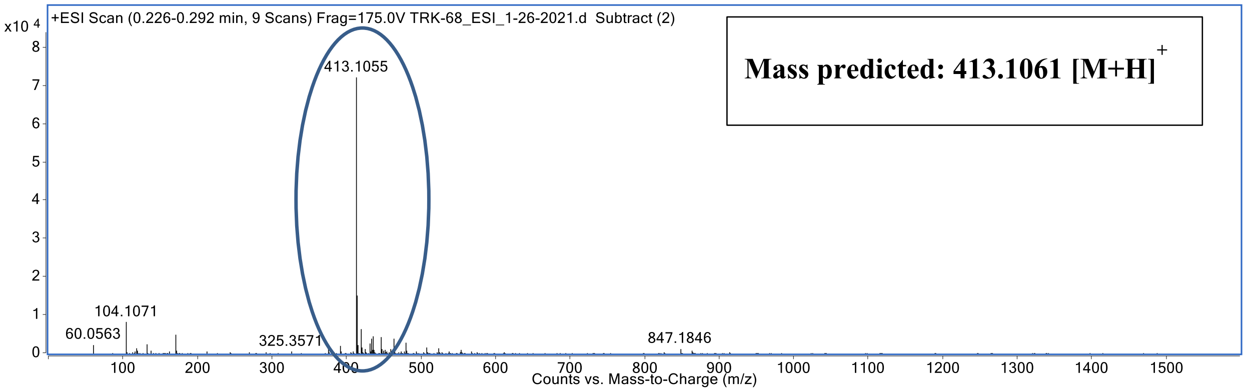

Supplemental Figure 46: HRMS of **11**
